# A Comprehensive Review on Irinotecan-Induced Delayed Diarrhea: From Mechanisms to Therapeutic Options

**DOI:** 10.3390/ph19081192

**Published:** 2026-07-29

**Authors:** Yanli Wang, Mengting Li, Qin Hu, Jieyuan Liu, Qing Yang, Qi Li, Xiaoxin Zhu, Haixia Dang, Hongmei Li, Lan Wang, Bo Peng, Ying Chen

**Affiliations:** 1 State Key Laboratory for Quality Ensurance and Sustainable Use of Dao-di Herbs, Institute of Chinese Materia Medica, China Academy of Chinese Medical Sciences, Beijing 100700, China; wyl656261@163.com (Y.W.); 18386078249@163.com (M.L.); m15665760697@163.com (J.L.); qyang@icmm.ac.cn (Q.Y.); qli@icmm.ac.cn (Q.L.); xxzhu@icmm.ac.cn (X.Z.); lihm2006@sina.cn (H.L.); lwang@icmm.ac.cn (L.W.); 2 College of Chemistry and Life Science, Beijing University of Technology, Beijing 100124, China; hq07616@bjut.edu.cn; 3 China Academy of Chinese Medical Sciences, Beijing 100700, China; danghaixia@126.com

**Keywords:** irinotecan-induced diarrhea, mechanism of action, enzymes, transporter, intestinal microenvironment, traditional Chinese medicine, clinical studies

## Abstract

Irinotecan is a first-line medication for metastatic colorectal cancer. It is known that irinotecan-induced delayed diarrhea is closely related to metabolic processes in the human body. In particular, the synergies of multiple metabolic enzymes/transporters mediate the transport/regeneration of intestinal SN38. This efficient metabolite derived from irinotecan not only exerts robust cytotoxic effects, but also extends contact duration with the intestinal wall during enterohepatic circulation. These factors further affect the intestinal microenvironment, including intestinal flora disorder, intestinal immune dysregulation, and intestinal barrier impairment, which ultimately causes the occurrence of delayed diarrhea. At present, dose adjustment of irinotecan and combining drugs are common measures for clinical use. However, these regimens have shown limited therapeutic efficacy and face the risk of chemotherapy failure. Numerous studies have shown that herbal medicines and derived phytochemicals may also be promising complementary treatments. The characteristics of multi-target regulation of herbs are very suitable for the intervention strategy of complex diseases with multiple pathogenic links. This review focuses on the development of irinotecan-induced delayed diarrhea. We generalized the complex pathogenesis in detail and summarized relevant potential drugs and clinical agents. Special attention was paid to the feasibility of traditional Chinese medicine treatment. This will help expand medication regimens for irinotecan-related diarrhea and provide a reference for the development of precision drugs.

## 1. Introduction

Chemotherapy is recognized as the current major treatment strategy for tumors. Nevertheless, non-specific cytotoxicity also causes inevitable damage to normal cells [1]. Gastrointestinal toxicity is a result of chemotherapeutic agents targeting gastrointestinal epithelial cells. It is reported that 40% of patients receiving chemotherapy experience gastrointestinal toxicity with standard doses, while those receiving higher doses are at greater risk of exposure, with the incidence ranging from 60% to 100% [2,3]. Clinically, gastrointestinal toxicity is characterized by various symptoms, including nausea, vomiting, anorexia, and diarrhea. Diarrhea is particularly notable for its high incidence rate (approximately 50–80%) and can be a serious cause of death [3,4,5,6]. According to the diarrhea grading by the National Cancer Institute, grade 3 or higher diarrhea requires more active medical intervention, including adjustment/interruption of the chemotherapy regimen, along with symptomatic treatment and nutritional support.

Irinotecan (CPT-11) is considered the first therapy choice for metastatic colorectal cancer and various malignant solid tumors. However, the incidence of diarrhea exceeding 60% causes limited clinical utility [7]. Notably, the incidence of severe (Grade 3 or higher) diarrhea induced by CPT-11 can range from 22% to 44%, potentially leading to severe complications such as dehydration, electrolyte imbalance, malnutrition, or cardio-renal dysfunction, thereby posing a life-threatening risk to patients [8]. CPT-11-induced diarrhea includes early-onset and delayed diarrhea. Early-onset diarrhea, typically defined as occurring within 24 h after administration, is caused by the transient inhibition of acetylcholinesterase. It can be promptly alleviated with anticholinergic drugs, such as atropine or scopolamine [9]. Delayed diarrhea, which typically occurs more than 24 h after administration, is characterized by its prolonged duration and greater severity. Therefore, close monitoring by clinicians and patients is required during CPT-11 treatment. It is widely accepted that the metabolism and elimination of CPT-11 dominate the occurrence of delayed diarrhea. As a prodrug, CPT-11 undergoes various metabolic transformations to form APC, NPC, SN-38, and SN-38G. Among these metabolites, SN-38 is responsible for the anticancer activity and exhibits thousands of times higher cytotoxicity than CPT-11 through contact with intestinal epithelial cells, making it the primary cause of delayed diarrhea [10].

Given the high incidence and high-risk characteristics of CPT-11-induced diarrhea, multiple preventive and interventional strategies have been proposed to date, including dose modification, formulation optimization, and combination therapy [10]. Regimen adjustment reduces intestinal exposure and accumulation of SN-38 by optimizing the dosage and administration frequency of CPT-11. Nevertheless, a balance between anti-tumor efficacy and intestinal toxicity must be maintained when adopting this strategy [10]. Formulation optimization improves the tumor selectivity of agents via targeted delivery systems, including liposomes, nanoparticles, and polymer–drug conjugates, thereby alleviating systemic adverse effects. Although favorable therapeutic potential has been demonstrated in in vitro preclinical studies, prominent limitations remain regarding the in vivo validation and clinical trial outcomes of most delivery carriers [11]. Combination therapy is regarded as a viable intervention strategy. Accordingly, guidelines issued by the European Society for Medical Oncology (ESMO) recommend loperamide and octreotide for the management of chemotherapy-induced diarrhea (CID) [6,12,13]. Nevertheless, loperamide administered at standard therapeutic doses often fails to effectively control moderate to severe diarrhea associated with CPT-11 [14]. Excessive doses of loperamide tend to trigger additional health hazards, including potential dependence and addiction, and cardiac arrhythmias [15,16,17,18,19]. Octreotide generally serves as the first-line alternative for patients unresponsive to loperamide. In clinical practice, frequent adverse events markedly elevate the failure rate of this therapeutic regimen [20]. Statistical data reveal that over 10% of patients discontinue octreotide-based diarrhea management due to concomitant adverse reactions, including hyperkalemia, neutropenia, nausea, abdominal discomfort, and arrhythmia [20,21,22]. In recent years, the evidence and advantages of TCM in treating CPT-11-induced diarrhea have gradually been revealed. First, in terms of therapeutic strategies, TCM allows for individualized treatment based on the clinical manifestations of patients [9]. Second, in terms of mechanisms, TCM often exerts synergistic actions. On one hand, it specifically intervenes with various metabolic enzymes and transporters involved in CPT-11 metabolism. On the other hand, it holistically regulates the intestinal microenvironment, including gut microbiota, intestinal immunity, and intestinal barrier functions [23].

The occurrence of CPT-11-induced diarrhea is the ultimate manifestation of changes in the intestinal microenvironment triggered by its metabolic outcomes within the body [6,10,11]. While TCM has exhibited promising efficacy in alleviating chemotherapy-mediated intestinal injury, the intrinsic connections between TCM interventions with multi-target advantages and CPT-11-induced diarrhea remain to be systematically elucidated. Accordingly, this review aims to understand the impact of the metabolic processes of CPT-11 on intestinal structure or function, further recapitulate the intervention mechanisms of preclinical/clinical agents, and focus on the therapeutic potential of TCM. The findings will provide guidance for the therapeutic direction of CPT-11 gastrointestinal toxicity and the development of precision medicines.

## 2. Method

### 2.1. Search Strategies

Three independent researchers systematically searched preclinical/clinical studies published between January 2000 and December 2025 for preventing/treating CPT-11-induced diarrhea. The search was conducted using Chinese databases (CNKI, VIP Database, and Wanfang Database) and English databases (PubMed, Web of Science, and Embase). Additionally, references in the studies were also considered to expand the search scope. The search keywords are set as: (herb OR Chinese herb OR herbal medicine OR Chinese medicine OR Chinese drug OR traditional Chinese medicine OR TCM) AND (CPT-11 OR irinotecan OR diarrhea OR chemotherapy-induced diarrhea OR irinotecan-induced diarrhea OR irinotecan-induced colitis OR CPT-11-induced colitis OR irinotecan-induced intestinal toxicity OR CPT-11-induced intestinal toxicity) AND (mechanism OR mechanism of action OR metabolic enzymes OR transporters OR gut microbiota OR intestinal flora OR intestinal barrier OR intestinal immunity OR intestinal inflammation OR metabolites).

### 2.2. Inclusion and Exclusion Criteria

The inclusion criteria were established as follows: (1) TCM formulas, extracts, or monomers were used as the primary intervention. Studies administering the herbal agents prophylactically prior to irinotecan chemotherapy or therapeutically after the onset of intestinal adverse reactions are both eligible for inclusion. (2) The enrolled clinical study subjects were patients receiving irinotecan-containing chemotherapy regimens (including irinotecan monotherapy, FOLFIRI, IFL, XELIRI, IROX, IP-NDP, IP, IC, mXELIRI, CapIRI, and FOLFOXIRI) who were diagnosed with diarrhea after chemotherapy. No restrictions are imposed on tumor type, patient gender, age, or diarrhea grade. (3) Preclinical studies, including in vivo animal experiments and in vitro cell assays, are eligible. Such studies must report sufficient quantitative pharmacodynamic indicators, primarily including diarrhea scoring, intestinal pathological injury evaluation, expression levels of inflammatory factors, quantitative detection of intestinal barrier markers, and measurements of intestinal immune function. The data should sufficiently verify that Chinese herbal compounds, extracts, or monomers alleviate irinotecan-induced diarrhea, intestinal mucositis, and systemic intestinal toxicity. No limitations are set on the strain, body weight, or age of experimental animals. (4) All preclinical basic experiments investigating the protective mechanisms against irinotecan-mediated intestinal injury are included. Relevant mechanistic research covers the regulation of metabolic enzymes and transporters involved in irinotecan biotransformation, as well as modulatory effects on intestinal physiological functions, such as intestinal barrier integrity, gut microbiota and their metabolites, intestinal immunity, and intestinal inflammation.

The exclusion criteria were established as follows: (1) Articles lacking pharmacodynamic test data, with no complete quantitative pharmacodynamic data available for statistical analysis in either the main text or Appendix A. (2) Duplicate records retrieved from multiple databases, with only the version containing the most complete research data retained. (3) Articles for which the full text could not be obtained after comprehensive searches of both the source databases and the corresponding official journal websites. (4) Studies that did not clearly state the dosage, administration frequency, or treatment duration of irinotecan or the tested Chinese herbal medicine. (5) Studies with ambiguous research design and data descriptions, from which pharmacological mechanisms related to intestinal protection cannot be inferred based on the pharmacodynamic results reported in the study.

### 2.3. Data Screening and Extraction

The entire process of literature identification, screening and inclusion in this study was performed in strict accordance with the PRISMA 2020 guidelines [24]. The initial literature records covered all entries retrieved from databases and clinical trial registration platforms. EndNote software was used to remove duplicate records, followed by primary screening based on titles and abstracts. Full-text rescreening was conducted on publications with accessible full texts according to the predefined inclusion and exclusion criteria. For relevant studies registered on clinical trial platforms (including randomized controlled trials and real-world studies), literature quality assessment and screening were jointly completed by researchers specializing in pharmacology and medical statistics.

The following information was uniformly extracted from each included study: (1) Basic bibliographic information: article title, publication year, and first author. (2) Quantitative pharmacodynamic indicators related to irinotecan-induced intestinal injury: diarrhea score, grading evaluation of intestinal pathological damage, expression levels of inflammatory factors, quantification of intestinal barrier-related proteins, and detection results of intestinal immune function. (3) Intervention regimen details: intervention agents, administration routes, and intervention duration. (4) Pharmacological mechanism data related to intestinal protection. The relative research concerning irinotecan metabolism-related enzymes and transporters, intestinal barrier repair, changes in gut microbiota and their metabolites, intestinal immunomodulation, and regulation of intestinal inflammatory pathways.

### 2.4. Search Results

**Figure 1:**
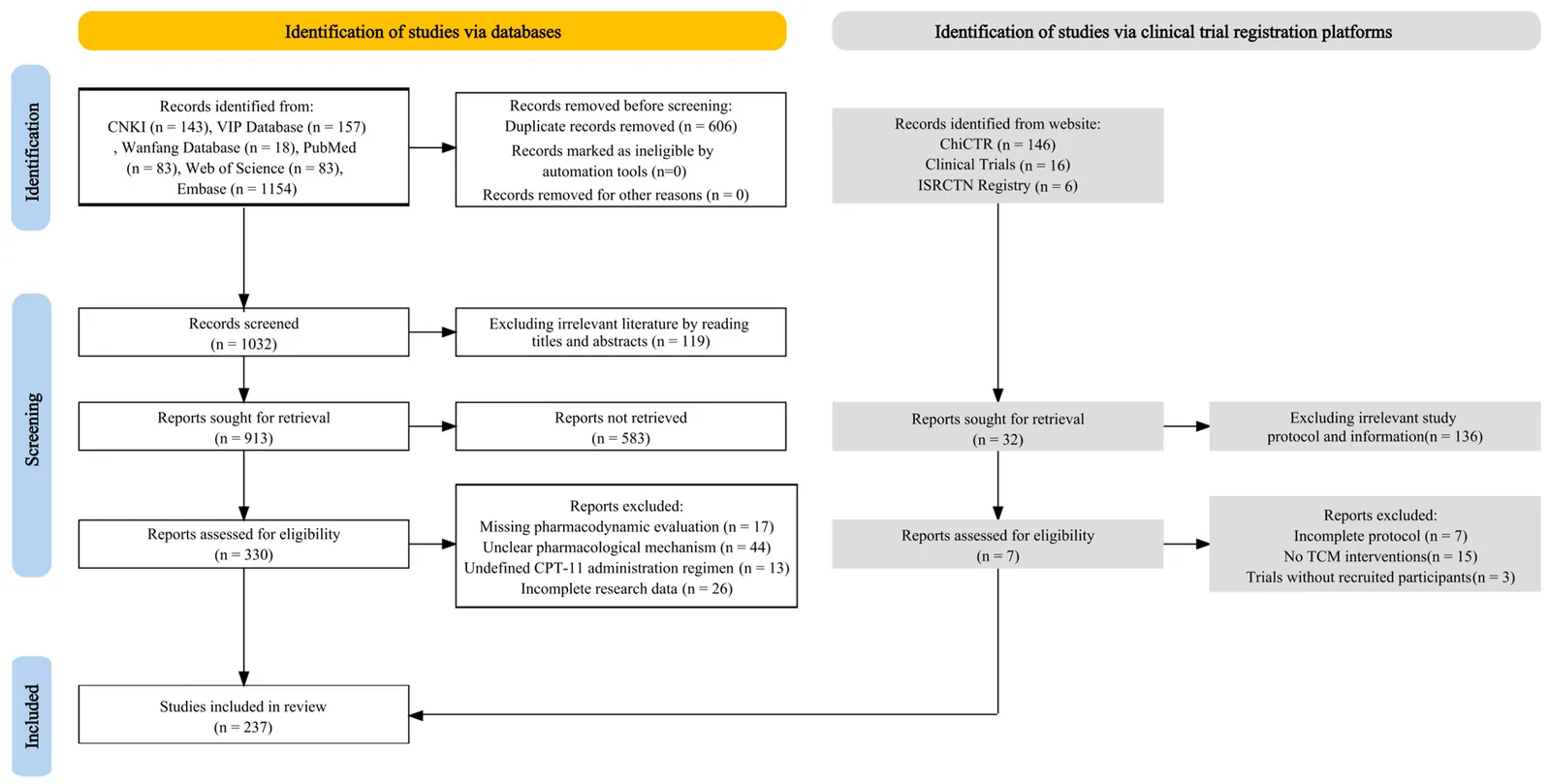
PRISMA flow diagram of literature screening process.

The initial literature search yielded a total of 1806 records. After removing 606 duplicate records using EndNote X9.3.3 software, 1200 records remained. Following screening by title and abstract, 255 irrelevant records were excluded, leaving 945 records for full-text assessment. After a thorough full-text review and stepwise screening against our predefined inclusion and exclusion criteria, a total of 237 studies were ultimately included in this systematic review. The complete literature screening process is illustrated in the PRISMA flow diagram (Figure 1).

## 3. CPT-11 Metabolism

**Figure 2:**
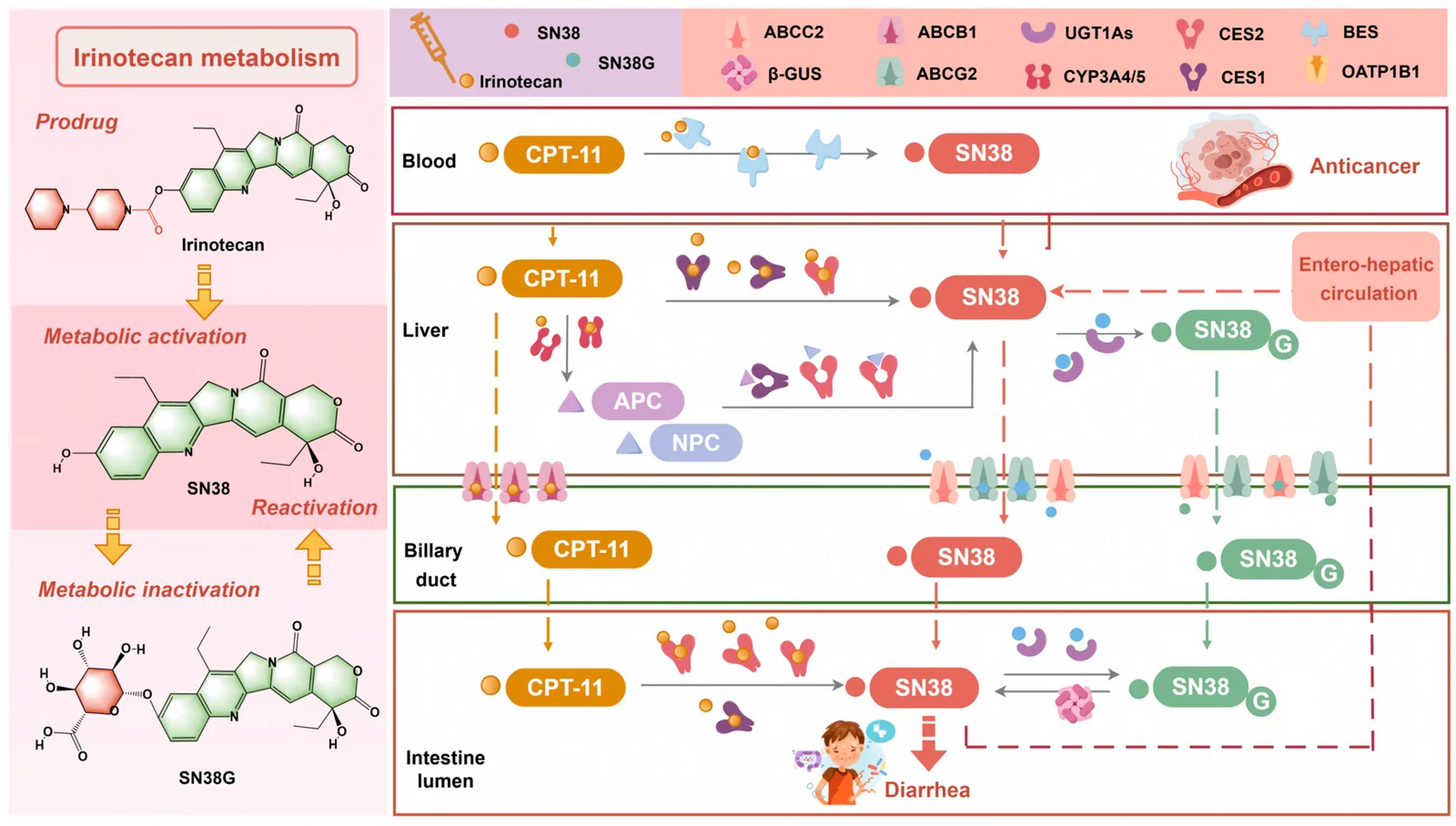
Schematic diagram of the in vivo metabolic transformation and enterohepatic circulation of CPT-11. Following intravenous administration, CPT-11 enters the systemic circulation and is taken up into hepatocytes via hepatic transporters (OATP1B1). In the liver, it is converted by carboxylesterases (CES) into the active metabolite SN-38, which is further glucuronidated by UGT1As to form SN-38G. CPT-11, SN-38, and SN-38G are excreted into the intestinal lumen via bile transporters (including ABCB1, ABCC2, and ABCG2). In the intestine, a portion of SN-38G is hydrolyzed back to SN-38 by β-glucuronidase, while intestinal CES2 can also convert CPT-11 into SN-38. CPT-11, SN-38 and SN-38G are reabsorbed through the intestinal epithelium into the portal vein, thereby re-entering the enterohepatic circulation.

As previously described, the in vivo process of CPT-11 involves multiple metabolic transformations, including the conversion from the prodrug (CPT-11) to the active metabolite (SN-38) and then to the inactive metabolite (SN-38G). Specifically, SN-38 amplifies the cytotoxicity of CPT-11. Its substantial accumulation in the intestine and prolonged contact with intestinal epithelial cells are key factors contributing to the occurrence of diarrhea [7,11]. In vivo conversion of CPT-11 primarily involves drug-metabolizing enzymes and transporters localized in the liver and intestine. A visual description is shown in Figure 2. Initially, CPT-11 is administered intravenously into the blood circulation. The CPT-11 and a small amount of SN-38 are taken up by hepatic transporters and transformed into SN38 and SN38G by metabolic enzymes. Subsequently, CPT-11, SN-38 and SN-38G are transported to the intestine via bile transporters. In the intestine, SN-38G is reconverted to SN-38 by the metabolic enzymes of the gut microbiota. Meanwhile, a portion of CPT-11, SN-38 and SN-38G is reabsorbed and participates in the entero-hepatic circulation for repeated transformation. In this section, we focus on the metabolism and disposition of CPT-11, with particular emphasis on the pivotal drug-metabolizing enzymes and transporters mediating the formation of SN-38.

### 3.1. Butyrylcholinesterase

Butyrylcholinesterases (BESs) are nonspecific esterases synthesized by the liver and primarily found in plasma. These enzymes exhibit multisubstrate specificity and can hydrolyze a variety of esters, peptides and amides [25,26]. Although BESs exhibit more than 6-fold higher bioactivity than hepatic carboxylesterases, their slow and inefficient catalytic efficiency results in a minimal contribution to the metabolic activation of CPT-11 [7]. To date, only limited studies have mentioned the involvement of BESs in the metabolic activation of CPT-11 in the blood, but the degree of their contribution has not been systematically evaluated [7,27].

### 3.2. Carboxylesterases

Carboxylesterases (CESs) are critical participants in the inactivation or detoxification of xenobiotics. Six CES subtypes have been identified, among which carboxylesterase 1 (CES1) and carboxylesterase 2 (CES2) are the main focuses in the study of drug metabolism [28]. Research reports have shown that CES1 and CES2 exhibit high sequence homology, yet they still present significant differences in distribution sites and substrate specificity [29,30,31]. It is widely accepted that human CES1 is highly expressed in hepatocytes, with minor expression in tissues such as the kidneys, monocytes, lungs, and intestines, whereas CES2 is predominantly distributed in the intestines and kidneys [32]. In clinical applications, CES1 tends to metabolize ester substrates containing small alcohols or large acyl groups, such as temocapril, oseltamivir, and sacubitri. In contrast, CES2 is responsible for the activation of antitumor prodrugs, including irinotecan and capecitabine [28]. For the metabolism of CPT-11, it is generally believed that CES2 has a higher binding affinity compared to CES1 (about 12.5-fold) and is the key enzyme for the conversion of CPT-11 to SN38 [33,34]. However, recent studies suggest that CES1 may have an equally important role as CES2 [29]. A study demonstrated that CES1 knockout mice displayed profoundly decreased conversion of the CPT-11 to SN-38. This reduction was effectively reversed to normal levels in a human CES1 transgenic mouse model, with SN-38 production increasing by 28.9-fold. These findings indicate that hepatic CES1 activity is a critical determinant for CPT-11 metabolism [35]. Compared with hepatic metabolism, intestinal CES2 has dual advantages in both binding affinity and quantity, which enable it to mediate more than 99% of the conversion of CPT-11 to SN-38 [34]. Currently, many scientists are focused on the development of potent CES2 inhibitors. It has been demonstrated that several small-molecule drugs, such as remdesivir and sofosbuvir, can inhibit CES2 activity and thereby reduce the formation of SN-38 in the intestine [36]. Additionally, plant-derived natural compounds, including baicalein and oroxylin A, have been shown to significantly reduce intestinal CES2 activity and exhibit inhibitory effects on the activation of CPT-11 [37,38].

### 3.3. Cytochrome P450

CYP3A4/5 is a secondary metabolic pathway for the formation of SN38. Under the catalysis of CYP3A4/5, CPT-11 is initially metabolized into the inactive metabolites APC and NPC, which are subsequently converted to SN38 by CES1 and CES2 in the liver [8]. Since this indirect transformation efficiency is much lower than the direct transformation of CPT-11, the demand for targeted drug development is limited. Scientists have shifted their focus to other investigations. Interestingly, Vander et al. successfully developed a CYP3A4 phenotype-based dosing algorithm for individualized treatment of irinotecan, which can significantly reduce inter-individual exposure variability of CPT-11 and SN-38 in clinical medication [39]. It provides potential for improving the predictability of CPT-11 pharmacokinetics and toxicity profiles, and offers a quantitative basis for the implementation of personalized CPT-11 treatment.

### 3.4. Uridine Diphosphate-Glucuronosyltransferase 1A1

Uridine diphosphate-glucuronosyltransferase 1A1(UGT1A1) is widely expressed in the liver and gastrointestinal tract. As the major glucuronic acid dehydrogenase, it is responsible for inactivating the lipophilic molecule SN-38 into the water-soluble metabolite SN-38G for elimination [7]. UGT1A1 gene polymorphisms are an important factor in reducing UGT1A1 enzyme activity and impairing detoxification pathways for CPT-11. In fact, its genetic polymorphisms have been utilized as biomarkers to predict the risk of diarrhea and adjust personalized CPT-11 regimen [10,40]. There are significant differences in UGT1A1 gene polymorphisms among different ethnic and regional populations. Among the over 100 known UGT1A1 polymorphic sites, the UGT1A1*28 and UGT1A1*6 genotypes have been reported to be most strongly associated with CPT-11-induced gastrointestinal toxicity [10]. The UGT1A1*28 genotype has been observed to have a high incidence of about 26–45% in African or Caucasian populations. In contrast, the UGT1A1*6 genotype is more prevalent in Asian populations, with an incidence of approximately 20% [7,10]. Given the significant impact of UGT1A1 genetic polymorphisms, numerous epidemiological studies have been conducted to optimize individualized medication dosing during CPT-11 use. Moreover, multiple national guidelines, including the Japanese Society for Cancer of the Colon and Rectum (JSCCR) Guidelines and European Society for Medical Oncology (ESMO) Guidelines, have explicitly proposed recommendations for CPT-11 use based on UGT1A1 genotyping. In terms of TCM therapy, Deng et al. found that ShengJiangXieXin Decoction could effectively reduce CPT-11-induced gastrointestinal toxicity without affecting clinical efficacy in oncological patients with UGT1A1*28 or UGT1A1*6 mutant phenotypes [41]. Silibinin has been shown to inhibit SN-38 glucuronidation catalyzed by UGT1A1*1 and UGT1A1*6, potentially leading to herb–drug interactions [42]. These findings provide valuable guidance for personalized treatments based on UGT1A1 genotyping.

### 3.5. β-Glucuronidase

β-glucuronidase (β-GUS), an intestinal bacterial enzyme, is recognized as a key mediator of chemotherapy-induced gastrointestinal toxicity in mammals. In CPT-11 metabolism, β-GUS is responsible for deconjugating SN38G to regenerate SN38. The increased intestinal exposure to this active metabolite is the fundamental cause of diarrhea. Accumulating evidence indicates that intestinal β-GUS activity is closely associated with the occurrence of dose-dependent diarrhea induced by CPT-11. In the rat model of CPT-11-induced diarrhea, an increase in the relative abundance of β-GUS-producing bacteria has been observed, along with enhanced β-GUS activity [43]. Similarly, clinical data have shown that β-GUS activity in patients with colorectal cancer is 12.1-fold higher than that in healthy individuals [44]. Targeting β-GUS-producing bacteria or inhibiting β-GUS activity are considered viable strategies to alleviate irinotecan-induced gastrointestinal toxicity. Several antibacterials (such as neomycin, cefixime, ciprofloxacin, streptomycin) and specific inhibitors (including amoxapine, TCH-3511, and TCH-3562) have been successively identified to mitigate irinotecan-mediated diarrhea [10,45,46,47,48,49,50]. For β-GUS inhibitors from natural products, Weng et al. identified that several natural flavonoids, including scutellarein, luteolin, baicalein, quercetin, and scutellarin, exhibit strong inhibitory activity [51]. Bei et al. demonstrated that berberine alleviates the intestinal toxicity by inhibiting β-GUS activity without affecting the antitumor efficacy of CPT-11 [52]. Additionally, several TCM extracts or formulas, such as green tea extract, ShengJiangXieXin Decoction, XiaoChaiHu Decoction, and DaBuPi Decoction, also showed positive inhibitory effects [53,54,55,56]. Although the gut is regarded as a major battleground for the pharmacological and toxic effects of CPT-11, most of the existing β-GUS inhibitors are designed to reduce the chemotherapy toxicity, with limited consideration of the impact on antitumor efficacy. Therefore, there remains a need to explore combination therapy strategies that target β-GUS without affecting or reducing the efficacy of CPT-11.

### 3.6. Organic Anion Transporting Polypeptides

Organic anion transporting polypeptides (OATPs), membrane transport proteins, exhibit broad tissue distribution and substrate specificity and are responsible for the pharmacokinetic processes of various drugs [57]. OATPs have several subtypes, among which OATP1B1 is specifically expressed on the hepatocyte basolateral membrane and is an essential protein for the uptake of SN38 in the liver [58]. In a study based on OATP1A/B1 knockout and humanized OATP1A/B1 transgenic mice, researchers robustly demonstrated that only SN-38 is a specific substrate for human OATP1B1 [59]. SLCO1B1, the coding gene of OATP1B1, has recently been shown to be highly genetically polymorphic, which may affect the in vivo exposure of SN38 and increase the risk of intestinal toxicity [58]. Similar to UGT1A1 gene polymorphisms, the distribution of SLCO1B1 gene polymorphisms also showed racial disparity. As the two most common mutation types, Mutation—388A>G and 521T>C of the SLCO1B1 gene have a high proportion in Europe and the Middle East (59.9% and 14.0%, respectively) [59]. In contrast, in Asian populations (such as China and Japan), the frequencies of these two mutations are approximately 70% for 388A>G and 14.0% for 521T>C, and the most common mutations were SLCO1B1* 1b and SLCO1B1*15 (59.9% and 14.0%, respectively) [60]. Given that SN-38 is responsible for both gut toxicity and anticancer activity, no targeted drugs have been used for SLCO1B1 gene polymorphism. Only a few TCMs have been reported to inhibit the cellular uptake of SN38 by OATP1B2, such as baicalin and capsaicin [61,62]. Although this may help reduce the risk of SN-38 accumulation in the gastrointestinal tract, further investigation is needed to evaluate the changes in the anticancer activity for clinical applications.

### 3.7. ATP-Binding Cassette Transporters

Biliary excretion is the major elimination pathway for CPT-11 and its metabolites. ATP-binding cassette transporters (ABC), including ABCB1 (encoding P-gp), ABCC2 (encoding MRP2), and ABCG2 (encoding BCRP), are responsible for transporting these substances into bile for excretion [10]. In transport selectivity, ABCB1 is the major efflux transporter that promotes biliary excretion of CPT-11 and facilitates the permeation of CPT-11/SN38 through the blood–brain barrier [63]. However, ABCC2 and ABCG2 are only involved in the efflux of SN38 and SN38-G [60,63]. In view of these transport properties, various drug evaluations were performed to find effective combination strategies. Verapamil, cyclosporine A, and thalidomide have been demonstrated to be potent inhibitors of ABCB1 [64,65,66]. Probenecid and thalidomide are considered potential candidates for reducing the expression of ABCC2 in the liver, thereby inhibiting the biliary excretion of SN-38/SN-38-G [66,67]. In addition, some TCMs, including quercetin, hesperidin, and XiaoChaiHu Decoction, have been reported to modulate the transporters involved in the metabolism of CPT-11 [68,69,70,71,72]. Among them, XiaoChaiHu Decoction has been highlighted for its ability to relieve CPT-11-induced diarrhea while preserving its antitumor efficacy, making it an ideal candidate for combination therapy with CPT-11. Recent studies have focused on the association between ABC gene polymorphism and CPT-11-associated adverse reactions (involving neutropenia and diarrhea). However, research on pharmacotherapeutic strategies targeting these genetic variations remains limited.

In summary, we have reviewed the metabolic enzymes and transporters directly involved in the disposition of CPT-11. This will facilitate a better understanding of the mechanisms underlying CPT-11-induced toxicity, aid in the identification of combination drugs with targeted features, and support the implementation of safer personalized clinical treatments. Nevertheless, during combination therapy, it is still necessary to carefully weigh and emphasize the balance between the safety and efficacy of CPT-11.

## 4. Effects of CPT-11 and Related Metabolites on the Intestine

**Figure 3:**
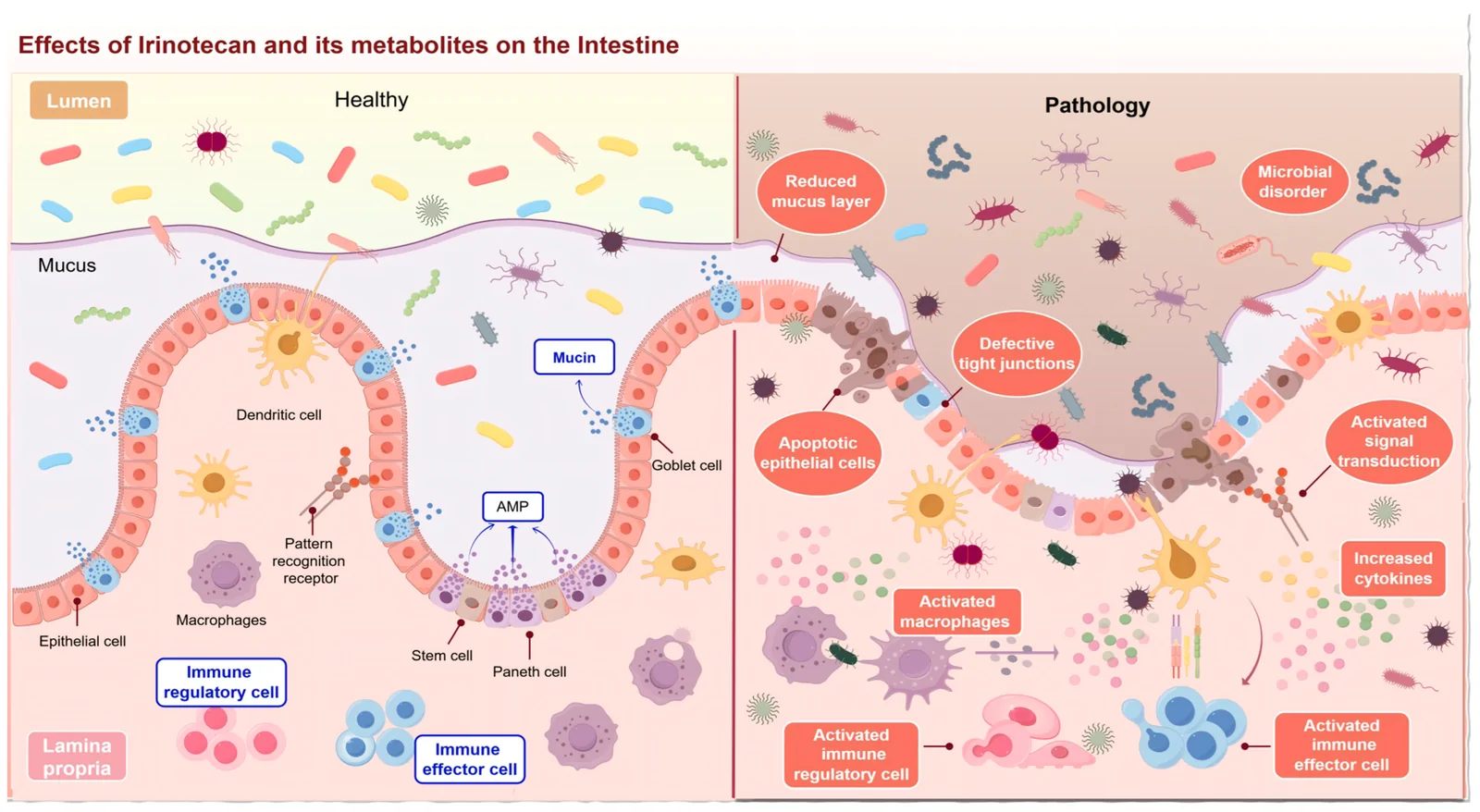
Changes in intestinal function under different physiological and pathological conditions. Compared with normal intestinal signaling and function, CPT-11 and its metabolites induce a series of intestinal functional impairments, including intestinal barrier disruption, gut microbiota dysbiosis, intestinal immune dysregulation, and the induction of intestinal inflammation. Among these manifestations, reduced mucus secretion, impaired tight junction protein function, and intestinal epithelial cell apoptosis represent the typical characteristics of intestinal barrier damage. Abnormal proliferation and differentiation of immune cells, coupled with the massive release of inflammatory factors, indicate abnormally activated intestinal immunity accompanied by the development of intestinal inflammation.

Previous reports have indicated that the biochemical mechanism of chemotherapy-induced diarrhea (CID) involves inflammation, secretion dysfunction, and functional gastrointestinal disorders [21]. As a typical chemotherapeutic agent, CPT-11 undergoes complex biotransformation in the body. A large amount of CPT-11 and SN38 accumulated in the intestine will successively induce alterations in intestinal structure and function, ultimately developing into delayed diarrhea [6,73]. In this section, we focus on summarizing the impact of CPT-11 and its metabolites on intestinal signaling and physiological functions, which is also illustrated in Figure 3.

### 4.1. Intestinal Barrier Impairment

The intact intestinal barrier can prevent hazardous foreign bodies (pathogenic microbes, endotoxin) from permeating through the intestinal mucosa, while facilitating the selective absorption of nutrients from the gut lumen. This functional barrier is collectively constituted by the mucus layer, intestinal epithelial cells, tight junctions (TJs), and the gut microbiota. In fact, the damaged intestinal barrier is a direct factor for CPT-11-induced diarrhea. The mucus layer serves as the first-line defense against the external environment/pathogens entering the gut lumen, which include mucin, antimicrobial peptides, lysozymes and other chemical components. Mucin 2 (MUC2), the most well-known mucin, is a major component of intestinal mucus. Abnormal changes associated with MUC2 production and secretion have been observed in a variety of inflammatory bowel diseases (such as ulcerative colitis, Crohn’s disease) [74,75,76]. Gibson et al. identified colon injuries caused by changes in goblet cells and mucin secretion during CPT-11-induced diarrhea [77]. Alterations in MUC gene expression were observed in the small intestine, demonstrating that accelerated mucus secretion and release may also contribute to CPT-11-related diarrhea [78]. However, another study reported that CPT-11 causes an increase in mucin secretion and a net decrease in mucin-producing goblet cells, which may be explained by changes in intestinal MUC2 and MUC4 expression [79]. The intestinal mucosal epithelium is another important factor in maintaining gut barrier integrity. The critical role of intestinal stem cells (ISCs) in intestinal epithelial regeneration has been well established, which is achieved through their self-renewal and differentiation into multiple types of intestinal epithelial cells [80]. A healthy intestinal mucosal epithelium is composed of a variety of intestinal epithelial cells and intercellular complexes, which serve as the important pathways for the transcellular transport/transepithelial paracellular leak of drug molecules/nutrients [81]. Previous studies have suggested a strong positive correlation between persistent intestinal epithelial cell damage and increased intestinal permeability [82]. CPT-11 and SN38 impair the self-renewal and repair of the intestinal epithelium by inhibiting the proliferation and differentiation of ISCs [83,84]. Such as in the cytotoxicity assessment of CPT-11 and SN-38 on intestinal epithelial cells, researchers have identified typical features of accelerated cell apoptosis and inhibited cell proliferation [85]. Moreover, several studies have similarly demonstrated the inevitable link between a damaged intestinal epithelial barrier and increased intestinal permeability associated with CPT-11-induced diarrhea [86,87]. In addition to the direct damage to functional intestinal epithelial cells, TJ proteins, which maintain the normal function of the paracellular pathway, also play a vital role in supporting the intestinal physical barrier [88]. The intestinal TJs are composed of various junctional molecules, including claudin, occludin, and ZO-1. Under steady-state conditions, TJs selectively permeate ions and hydrophilic small molecules to prevent harmful macromolecules or gut microbiota from entering the body [89]. However, following CPT-11 exposure, the gene expression and protein levels of claudin, occludin, and ZO-1 are significantly reduced. This defect in TJs promotes intestinal mucosal barrier dysfunction, gut bacterial translocation and the development of diarrhea [90,91].

### 4.2. The Disturbance of Gut Microbiota and Metabolites

The role of the gut microbiota in maintaining host health cannot be overlooked. Its symbiotic balance with the host is beneficial for enhancing immune activity, promoting intestinal metabolism, and reducing pathogen susceptibility [92]. Brandi et al. found that germ-free mice were more resistant to CPT-11-induced diarrhea than holoxenic mice, which confirmed that intestinal microbiota played a key role in the intestinal toxicity of irinotecan [93]. Currently, the effects of CPT-11 on gut microbes can be summarized into two categories. A significant change was observed in the structure of the gut microbiota in rats treated with CPT-11, with a notable reduction in beneficial bacteria such as Lactobacillus and Bifidobacterium [43]. Mahdy et al. further investigated the differences in gut microbiota derived from clinical fecal samples, and found that the abundance of *Actinobacteria*, *Verrucomicrobia*, *Bifidobacterium* and *Phascolarctobacterium* was significantly decreased in the CPT-11 group, which may induce the increased intestinal inflammation [94]. In addition to the impact on microbial composition/structure, recent studies have also highlighted that the CPT-11-mediated intestinal toxicity may also be a consequence of intestinal flora metabolites. Gut microbial metabolites not only serve as key mediators of intestinal immune regulation but also enhance the gut barrier through bidirectional interactions with intestinal epithelial cells [95,96]. These metabolites can be categorized into several classes, including derivatives from exogenous products (ginsenoside CK, berberine, and protopine), de novo synthesized metabolites (short-chain fatty acids and indole derivatives), and metabolites initially produced by the host and secreted into the lumen, which are then modified by gut microbes (secondary bile acids) [96]. CPT-11 primarily disrupts the metabolic functions of the latter two categories. Short-chain fatty acids (SCFAs), key metabolites derived from gut microbial fermentation, primarily include acetate, propionate and butyrate. They serve as critical mediators of host health by providing energy to intestinal epithelial cells and maintaining gut barrier integrity [97]. CPT-11 can affect SCFAs in several aspects. Notably, Bacteroides, Akkermansia and Bifidobacterium, previously reported as dominant contributors to SCFA production [97,98,99,100], exhibit significantly reduced abundance in CPT-11-induced gut dysbiosis, thereby impeding SCFA synthesis [54,101]. Furthermore, CPT-11 and SN38 accumulated in the intestinal lumen will increase intestinal toxic load, forcing increased energy requirements for epithelial repair and regeneration processes, which further depletes the content of SCFAs, especially acetate and butyrate [102]. Ultimately, CPT-11-mediated SCFA alterations promote intestinal hyperpermeability, which is characterized by decreased secretion of MUC2, severe intestinal epithelial dysfunction, and reduced expression of tight junction proteins [10,54]. Wang et al. revealed the alterations of endogenous metabolites related to CPT-11 perturbation, including abnormal tryptophan and bile acid metabolism [103]. Tryptophan metabolism is known to be an important contributor to intestinal homeostasis, and its metabolites are able to affect host function, such as indole acetic acid (IAA), indole formaldehyde (IAld) and indole acrylic acid (IA), and indole lactic acid (ILA) [104]. CPT-11 impairs tryptophan metabolism, which tips the balance in the gut immune microenvironment maintained by IAA/IA/IAld/ILA-AHR activation. The most typical change is the altered differentiation of CD4+ T cells into Th17 and Treg cells [104,105]. Meanwhile, recent studies have highlighted that AHR activation induced by indole derivatives is also beneficial in defending intestinal mucosal homeostasis. Specifically, the activated IAId-AHR-IL-22 pathway has been proven to effectively reduce mucosal damage in mice [106]. Furthermore, a prospective cohort study revealed that *B. intestinalis* enriched after CPT11 chemotherapy exacerbates irinotecan-induced intestinal epithelial injury through the tryptophan metabolite indole-3-acetic acid (IAA), providing a novel strategy for predicting individualized toxicity [107]. Bile acids (BAs) are another class of microbial metabolites that fulfill fundamental physiological functions. They are initially converted from cholesterol in the liver to primary BAs and stored in the gallbladder. This is subsequently released into the intestinal lumen in response to the host demands and modified by gut microbes to form secondary BAs for physiological functions [108]. As a signaling mediator, the Bas-FXR/TGR5 pathway is one of the effective regulatory pathways in BA signaling [109,110,111]. Previous studies have shown that CPT-11 may affect bile acid homeostasis, which could trigger FXR/TGR5-related signaling cascades to modulate gut barrier function or control inflammation [110,112]. Additionally, in the aspect of immunomodulation, Fang et al. demonstrated that CPT-11-induced bile acid metabolism disorders aggravated suppression of IL-10 expression, leading to severe intestinal inflammation in mice [113].

### 4.3. Intestinal Immune Dysregulation

The immune system is responsible for recognizing harmful threats and mobilizing autonomous defense capabilities. As the largest immune organ, the gut harbors a dense population of immune cells within the epithelial layer, lamina propria, and gut-associated lymphoid tissues, which collectively serve to counteract potential threats [114]. As previously described, we have summarized the intestinal dysfunction caused by CPT-11 and its metabolites, which includes disruption of the gut barrier (physical barrier, chemical barrier, and microbial barrier). This disruption may be one of the causes of the altered immune microenvironment. Bacterial translocation is a typical feature of a CPT-11-impaired barrier [115,116]. Studies have observed that the continuous damage to the intestinal epithelial cells caused by CPT-11 allows Gram-negative bacteria to permeate into the intestinal lamina propria, thereby activating the signal transduction and inflammatory cytokine release of intestinal immune cells (macrophages, neutrophils) [117]. Recently, a study was conducted to explore the correlation between gut microbiota and human immune cells, and it was identified that *Faecalibacterium*, *Ruminococcus*, and *Akkermansia* are the most potent intestinal microbiota in driving immune responses and functions [118]. However, CPT-11 leads to decreased relative abundances of *Ruminococcus* and *Akmansia* in the intestinal tract [119], which further supports the evidence that CPT-11 can influence gut immune function. In addition to the immune dysfunction mediated by CPT-11-induced barrier disruption, the direct cytotoxicity from CPT-11 and SN-38 can accelerate the apoptosis of intestinal crypt cells. The self-derived damage-associated molecular patterns (DAMPs) can be recognized by pattern recognition receptors (PRRs) on immune cells, such as Toll-like receptors (TLRs) and NOD-like receptors (NLRs), thereby promoting the release of inflammatory cytokines. He et al. demonstrated that CPT-11 can induce abnormal innate immunity by modulating TLR signaling in fruit flies [120]. Li et al. found that CPT-11 can activate the NLRP3 inflammasome in macrophages to release IL-1β/IL-18, which contributes to the occurrence of diarrhea [121]. Moreover, persistent noxious stimuli further initiate adaptive immune responses. Xue et al. first elucidated the effects of CPT-11 on systemic and intestinal immunity in tumor-bearing rats, and found that CPT-11 promoted activation of intestinal T cells while inducing hyporesponsiveness of spleen cells [122]. Fernandes et al. emphasized the regulatory role of Treg cells in CPT-11-induced intestinal mucositis and found that Treg cell depletion broke the original immune balance, leading to uncontrolled inflammatory responses, which aggravated intestinal injury, diarrhea progression, and neutrophil infiltration [123].

### 4.4. Intestinal Inflammation

The intestinal microenvironment represents a potentially hostile environment. It not only has resistant defense related to the intestinal barrier, but the inflammatory response can also serve as a protective measure for the body to eliminate harmful threats and repair damaged tissues [124]. Upon sensing potential threats, the body initially forms a positive feedback loop based on “immune cells-cytokines “regulation. However, once this regulatory mechanism fails, uncontrolled excessive inflammation will persistently cause tissue damage [125]. A recent study reported that various endotoxins and exotoxins possess an extremely potent ability to induce inflammatory cytokines (TNF-α, IL-1β, IFNγ), which is a cascade effect of multiple signal transduction [126]. This finding explains the accumulation of inflammatory cytokines following CPT-11 administration. On one hand, CPT-11 can cause bacterial translocation, increasing the contact probabilities between bacterial endotoxins and macrophages. On the other hand, CPT-11 or SN-38 acts as a cytotoxic agent, exerting an exotoxin-like induction effect. As previously mentioned, the continuous disruption of CPT-11-caused intestinal epithelial cells allows Gram-negative bacteria to penetrate into the intestinal lamina propria, leading to the production of large amounts of bacterial endotoxins (including lipopolysaccharides). This subsequently results in the release of inflammatory cytokines associated with TLR4-NF-κB/JNK signaling [10]. For the exotoxin effect of CPT-11, studies have observed severe intestinal lesions and excessive secretion of pro-inflammatory cytokines (IL-1β, IFN-γ, and TNF-α) in a rat model of CPT-11-induced diarrhea [127,128]. Transient receptor potential ankyrin 1 (TRPA1), a sensor for cellular stimulation, has been demonstrated to be closely associated with the intestinal inflammation induced by CPT-11. Inhibition of TRPA1 can mitigate intestinal inflammatory damage, reduce the release of inflammatory markers (TNF-α, IL-1β, MPO, NF-κB, and COX-2), and decrease the levels of oxidative stress (GSH, MDA, and NOx) [129]. Additionally, the NLRP3-IL1β inflammasome pathway is also a key factor in CPT-11-induced diarrhea [10,121].

In conclusion, the intestinal damage induced by CPT-11 is attributed to multiple factors, including intestinal inflammation, immune dysregulation, and barrier dysfunction. The current summary helps elucidate the specific pathways by which CPT-11 affects intestinal function and identifies potential biomarkers associated with these pathways. This provides a systematic insight into the diagnosis and treatment of CPT-11-induced diarrhea.

## 5. Therapeutic Interventions for CPT-11-Induced Diarrhea

Based on the aforementioned interaction between CPT-11 and the host, we found that multi-target intervention strategies were more effective in reducing the incidence of diarrhea caused by CPT-11. In this section, we first review the existing clinical management approaches, followed by an overview of clinical/preclinical studies on targeted therapeutic candidates, with particular emphasis on the role and mechanisms of TCM.

### 5.1. Clinical Management

CPT-11 is classified as a chemotherapeutic agent, and the intestinal toxicity monitoring primarily relies on the management protocols for CID. Given that CID severity is closely related to the type, regimen, and dosage of chemotherapeutic agents, in order to define the CID levels and develop a universal management protocol, the National Cancer Institute (NCI) has defined CID risk levels based on patients’ defecation status and the degree of functional impairment in daily activities. These risk levels are detailed in the latest Common Terminology Criteria for Adverse Events (CTCAE, Version 5.0), which outlines the corresponding manifestations for diarrhea grades (from Grade 1 to Grade 5) [130]. In addition, the guidelines issued by the European Society for Medical Oncology (ESMO) correlate the aforementioned diarrhea grades with clinical symptoms (including fever, gastrointestinal bleeding) to define the degree of CID as severe/non-severe diarrhea. They also propose recommended medications such as loperamide and octreotide [131]. Loperamide, an opioid receptor agonist, can effectively control diarrhea by reducing intestinal motility, inhibiting intestinal secretion, prolonging intestinal retention, and enhancing anal sphincter tone [132]. Octreotide, a COX2 inhibitor, stimulates the thromboxane A2 to inhibit chloride and water secretion by intestinal epithelial cells and is widely used for severe CPT-11-caused diarrhea [131]. Although these drugs can effectively alleviate symptoms, they still pose potential risks when administered continuously during chemotherapy or when used to control diarrhea induced by high doses of CPT-11. For example, loperamide may induce paralytic ileus, while octreotide can cause arrhythmias, constipation, abdominal cramps and nausea. Moreover, clinical drugs such as thalidomide, phenobarbital, and budesonide have also been reported to modulate CPT-11 metabolism/the intestinal microenvironment to mitigate diarrhea. Xu et al. provided a detailed summary of their mechanisms of action, dosing regimens, and potential adverse effects [10]. It is evident that these current therapies are generally non-specific and target single-link regulation, exhibiting limited efficacy and inevitable adverse reactions when used to alleviate CPT-11-related diarrhea. In particular, the balance between safety and efficacy poses significant challenges for clinicians in prescribing these medications. Therefore, it is necessary to develop therapeutic strategies with fewer side effects or superior efficacy.

### 5.2. Preclinical Studies

Given the limitations of current clinical management strategies, the development of precision drugs involved in CPT-11-induced diarrhea remains a scientific objective of exploration. Here, we review the potential agents currently under preclinical investigation, including small-molecule chemical drugs and natural products derived from TCM.

#### 5.2.1. Small-Molecule Chemical Drugs

Small-molecule chemical drugs have consistently held a dominant position in the development of precision medicine. Some specific drugs that alleviate CPT-11-induced diarrhea are summarized in Table 1, and related regulatory pathways involve important metabolic enzymes/transporters, signal transduction receptors, and cytokines. LE-77, a small-molecule compound, was first identified and used to treat irinotecan-induced intestinal toxicity by Cao et al. They reported that LE-77 functions as a dual modulator to exert therapeutic effects. On one hand, it could inhibit intestinal CES2 activity to reduce intestinal exposure to SN-38. On the other hand, it helps to activate the FXR signaling receptor to play anti-inflammatory and protective effects on the intestinal mucosa [133]. Recombinant human interleukin-1 receptor antagonist (rhIL-1Ra) is a specific inhibitor of the cytokine IL-1. Wang et al. evaluated the protective effects of rhIL-1Ra in mice with CPT-11-induced mucositis, and found that it alleviated intestinal inflammation and crypt apoptosis by intervention of cell cycle, thereby effectively protecting intestinal epithelial cells from damage [134]. It is known that CPT-11-induced mucositis is associated with the activation of the innate immune receptor TLR4. A study has identified that IAXO-102 can attenuate CPT-11-caused gastrointestinal inflammation by selectively interfering with the TLR4 receptor. Recently, carbon adsorbents have also shown promising applications in the treatment of CPT-11-related diarrhea. AST-120, an oral adsorbent composed of porous carbonate particles, has a strong adsorption capacity for camptothecin drugs, which is beneficial to adsorb CPT-11 and SN-38 free in the intestinal lumen to reduce intestinal exposure and relieve diarrhea [135,136]. Undoubtedly, the discovery of small-molecule drugs has significantly expanded the treatment options for CPT-11-induced diarrhea. However, the current single-target intervention model still cannot meet the multi-pathway detoxification strategies required for treating CPT-11-induced diarrhea. Moreover, other safety concerns, especially the risk of hepatotoxicity induced by drugs targeting related metabolic enzymes/transporters of CPT-11, remain to be thoroughly evaluated.

#### 5.2.2. TCM from Natural Sources

**Figure 4:**
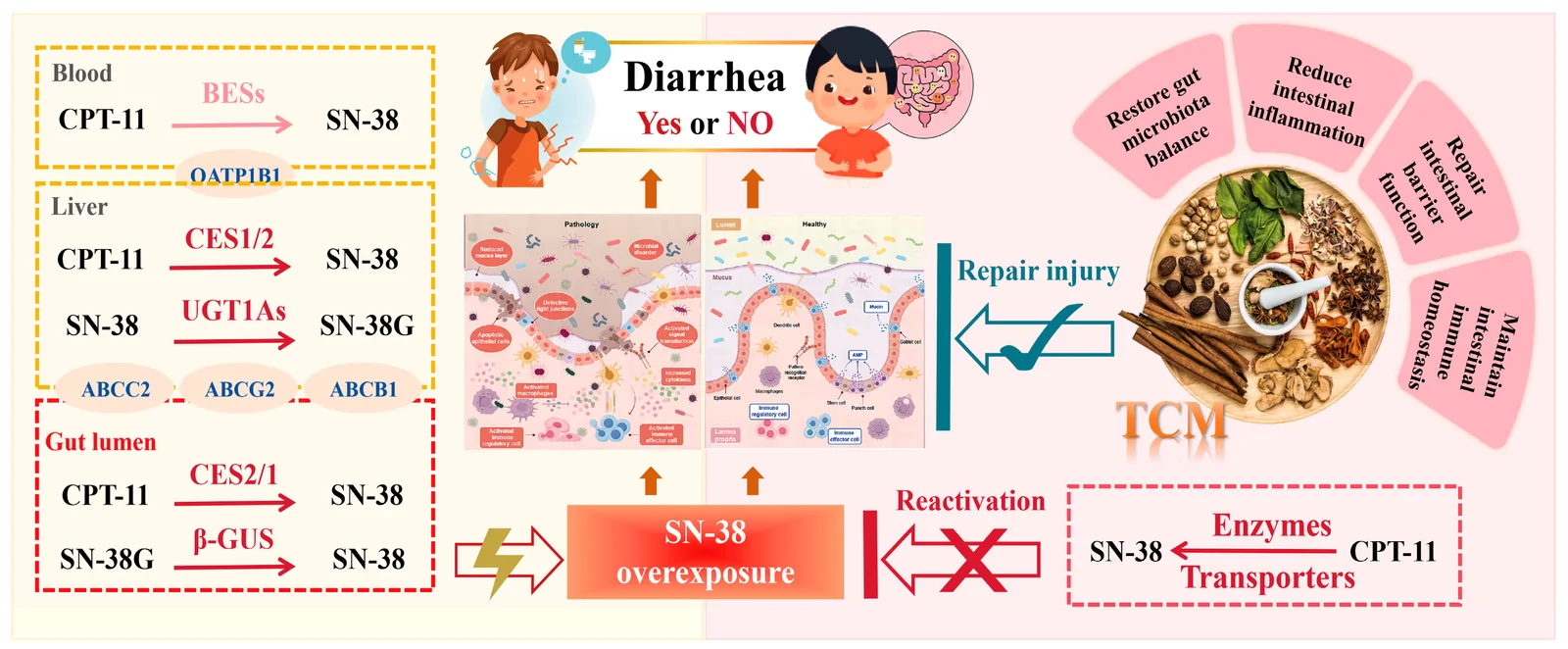
Potential regulatory pathways of TCM for irinotecan-induced diarrhea. Under the mediation of various metabolic enzymes and transporters, CPT-11 leads to excessive exposure of SN38 in the intestine, thereby aggravating intestinal burden and leading to a series of intestinal disorders. These include impaired barrier function, dysbiosis, metabolic disturbances, immune dysregulation, and inflammation, collectively contributing to diarrhea. TCM, characterized by its multi-target integrated regulatory properties, can exert holistic therapeutic effects by synergistically acting on the aforementioned pathological processes.

TCMs refer to medicinal substances or preparations applied under the guidance of TCM theory. Since ancient civilizations, TCMs have been utilized for health maintenance and disease treatment across Asian regions [146]. In recent years, TCMs have been recognized as complementary and alternative medicines in Western countries, and are commonly combined with other pharmaceuticals to achieve superior therapeutic efficacy and alleviate adverse side effects [27,146,147]. Evidence indicates that TCM formulas/extracts with multi-pathway synergistic effects may be a valuable source for developing multi-target therapies for complex diseases. As illustrated in Figure 4, TCM can exert regulatory effects at various stages of CPT-11 metabolism and intestinal function to different extents. In this section, we have systematically summarized the currently studied TCM formulations/extracts/compounds targeting these processes, as presented in Table 2. Meanwhile, we elucidate the underlying regulatory mechanisms using representative TCM examples.

##### Metabolic Enzymes/Transporters

The activity/expression of metabolic enzymes/transporters directly affects the in vivo exposure of CPT-11 and its metabolites. It has been reported that various TCMs can alleviate CPT-11-induced diarrhea by this pathway. ShengJiangXieXin Decoction (SJXXD), a classic formula composed of eight herbs, has been proven to be effective in treating bacterial or chemotherapy-related diarrhea/gastroenteritis [201]. Guan et al. found that this protective effect might be explained by the regulation of SJXXD on metabolic enzymes and transporters. This formula not only reduces the expression of Mrp-2 and P-gp in the liver to prevent the transport of CPT-11 and its metabolites through the “bile-intestine” pathway, but also increases the expression of intestinal UGTs to accelerate the metabolic conversion of SN-38 to SN-38G. This directly reduces the intestinal exposure to CPT-11/SN-38, thereby alleviating damage [71]. Similarly, XiaoChaiHu Decoction (XCHD) also reduces the intestinal toxicity by affecting the pharmacokinetics process of CPT-11 and its metabolites, which can be attributed to its inhibitory effects on efflux transporters (ABCB1, ABCC2, ABCG2) and intestinal β-GUS activity, while simultaneously increasing the expression and activity of intestinal UGTs. These factors result in impaired biliary excretion and reduced intestinal accumulation of CPT-11, SN-38, and SN-38G [72,160,162]. In addition, other TCM formulas or components, such as SiShen Pill, WeiChangAn Pill, green tea extract, berberine and genistein, also reduce the exposure of intestinal CPT-11 and SN38 through the metabolic enzyme/transporter pathway to exert anti-diarrheal effects [52,54,70,171,172].

##### Gut Microbiota

Intestinal flora is highly related to host health. In particular, the role of TCM in restoring intestinal microbial homeostasis to inhibit disease occurrence has been widely reported. It has been established that CPT-11 induces gut microbiota dysbiosis, characterized by an increased relative abundance of Bacteroides and a decreased relative abundance of *Lactobacillus*. *Pogostemon cablin*, a medicinal food homology plant, can significantly improve the symptoms of diarrhea caused by CPT-11. Li et al. further found that this efficacy may be attributed to the indirect effect of intestinal microbiota. Pogostemon cablin granules beneficially regulated intestinal microbial community structure, including reducing the relative abundance of the Bacteroides and increasing the relative abundance of the Lactobacillus, thereby restoring gut microbial homeostasis [169]. XCHD is widely used to alleviate CPT-11-induced diarrhea [161]. Wang et al. found that it has both holistic and specific regulatory advantages. Overall, this formula can modulate the structure of the gut microbiota, with a significant increase in the abundance of beneficial phyla, such as Firmicutes and Bacteroidetes. Moreover, baicalein, an active component in the XCHD, specifically binds to the microbial metabolite β-GUS to inhibit enzyme activity, thereby reducing intestinal exposure to SN-38 [53]. Similarly, glycyrrhiza total flavones can reduce the abundance of β-GUS-producing bacteria (such as *Escherichia coli* and *Clostridium* spp.) while increasing the abundance of SCFAs-producing bacteria (*Roseburia* spp.), effectively alleviating CPT-11-induced colitis [183].

##### Intestinal Barrier

As an essential defense system of the gut, the intestinal barrier is responsible for providing robust protection against internal and external pathogenic microorganisms. In recent years, the role of TCM in treating diseases related to gut barrier disruption has gradually garnered attention. PHY906, a standardized TCM formulation derived from HuangQin Decoction, was initially developed as an anticancer adjuvant to improve therapeutic indices and mitigate the side effects of various chemotherapeutic agents, including sorafenib, irinotecan, and capecitabine [202]. Owing to its promising anticancer activity and potential to ameliorate adverse reactions, PHY906 is currently being investigated in multiple clinical trials. In the improvement of CPT-11-induced diarrhea, studies have demonstrated the positive effects of PHY906 on regulating the intestinal barrier and inflammation. On one hand, PHY906 promotes intestinal cell regeneration via the Wnt signaling pathway to repair intestinal epithelial damage. On the other hand, it reduces the infiltration of neutrophils or macrophages to mitigate intestinal inflammation [151]. HuangLianJieDu Decoction (HLJDD), a TCM formulation composed of four herbs, is well-known for its anti-inflammatory, antioxidant, and antipyretic efficacies. Recent studies have explored its potential as an adjuvant therapy for CID. In CPT-11-induced diarrhea, HLJDD pre-treatment can maintain CD44 levels in renewing crypt cells, thereby improving the activity of the Wnt/β-catenin pathway to sustain rapid cell renewal, effectively preventing intestinal barrier damage [167]. Similarly, SJXXD promotes intestinal stem cell regeneration to maintain the normal function of intestinal epithelial cells [55].

##### Intestinal Immunity

It is known that intestinal immune dysregulation associated with CPT-11 also increases the risk of diarrhea, and several TCMs have been found to restore immune homeostasis. Deng et al. reported that SJXXD increased the CD4+ T/CD8+ T lymphocytes in the intestinal mucosa and enhanced the expression of secretory immunoglobulin A (SIgA), thereby resisting diarrhea associated with CPT-11-induced intestinal immune deficiency [55]. The aqueous extract of Prunus mume elevated the CD4/CD8 ratio in the colon, exerting positive immunomodulatory effects to alleviate diarrhea [187].

##### Intestinal Inflammation

Inflammation is a danger signal in various diseases. TCM can mitigate diarrhea by blocking the inflammatory cascade triggered by CPT-11 and reducing the production of inflammatory cytokines. GeGenQinLian Decoction has been reported to have a clinical value similar to that of HuangQin Decoction. Cheng et al. identified that its positive effects on inflammation inhibition and intestinal mucosal barrier repair are dependent on the PI3K/AKT/NF-κB pathway based on bioinformatics combined with experimental analysis [165]. TCM theory originates from the systematic summarization of clinical practices by ancient Chinese physicians. According to this theory and the properties of herbs, the ShuBuWenShenGuChang Formula is beneficial for inhibiting diarrhea and alleviating gastrointestinal pain. Zhang et al. investigated its efficacy against CPT-11-induced diarrhea. It was found that this formula inhibits the activation of the TLR4/NF-κB/NLRP-3 pathway to alleviate intestinal inflammation [173]. HeZhong Formula, a reconstituted TCM prescription based on the TCM theory and modern clinical practice, aims to relieve the gastrointestinal toxicity caused by chemotherapy in cancer patients. For CPT-11-induced diarrhea, Wang et al. found that the therapeutic mechanism may be related to the inhibition of AIM2 inflammasome activation and reduction of inflammatory cytokine secretion, which in turn decreases intestinal mucosal inflammation and cell apoptosis [174]. Additionally, well-known formulas such as BanXiaXieXin Decoction and ShengJiangXieXin Decoction have demonstrated their potential in blocking intestinal inflammation, providing evidence for their application in treating CPT-11-related diarrhea [154,156,158,203,204].

##### Intestinal Endogenous Metabolism

Endogenous metabolites play dual roles in both signal transduction and functional execution. The intervention of TCM in the perturbation of endogenous metabolites caused by CPT-11 may be an important reason for its protective effects. HuangQin Decoction, a typical traditional Chinese medicine compound, alleviates diarrhea by regulating amino acid metabolism (glycine, serine, threonine) and bile acid homeostasis [103,150]. SJXXD significantly increases the levels of SCFAs, which may contribute to maintaining the intestinal barrier integrity [155]. Glycyrrhizin total flavonoids have the potential to mitigate the fluctuations in amino acid metabolism and purine metabolism caused by CPT-11 [183]. While these findings provide strong support for TCM treatment strategies that rely on correcting CPT-11-induced metabolic disorders, further investigation is still required to clarify specific metabolites and regulatory pathways.

## 6. Clinical Studies of TCM

Although sufficient preclinical studies facilitate the acceleration of clinical translation. In current reports, we have found that there is only limited high-quality evidence-based data to systematically evaluate the effect of TCM in improving CPT-11 chemotherapy-induced diarrhea. In this section, we aim to evaluate typical clinical studies and predict the potential transformation of promising TCM with preclinical evidence.

### 6.1. Evaluation of Clinical Studies

In addition to the recommended therapeutic agents, certain TCM preparations have demonstrated significant efficacy in clinical evaluations. A recent meta-analysis reported that TCM interventions significantly reduced the overall incidence of CID (RR = 0.55, 95% CI = 0.40–0.75, *p* = 0.0002), with a particularly pronounced preventive effect on severe diarrhea (grade 3–4) (RR = 0.41, 95% CI = 0.26–0.64, *p* < 0.0001) [9]. We systematically reviewed clinical trials investigating TCM for the management of CPT-11-associated diarrhea, as shown in Table 3. Among the 19 completed clinical trials, BanXiaXieXin Decoction (10 trials) and HuangQin Decoction (3 trials) emerged as the primary interventions. These formulations not only significantly reduced the incidence of CPT-11-induced diarrhea but also demonstrated potential benefits in alleviating other adverse events, including mitigation of myelosuppression, improved nausea and vomiting, and reduced risk of neutropenia and thrombocytopenia. Meta-analysis data further confirmed that HuangQin Decoction significantly reduced the risk of diarrhea by 47% (RR = 0.56, 95% CI = 0.43–0.72, *p* < 0.00001), while BanXiaXieXin Decoction demonstrated an even more pronounced effect, reducing diarrhea risk by 71% (RR = 0.18, 95% CI = 0.05–0.63, *p* = 0.007) [9]. Furthermore, four interventional randomized controlled trials (RCTs) have been registered on clinical trial platforms, including ChiCTR, ClinicalTrials.gov, and ISRCTN Registry. These registered studies involve ShengJiangXieXin Decoction (ChiCTR1800018490), berberine (ChiCTR2300078353), and XiaoChaiHu Decoction (NCT04926545 and NCT06055179). Although the total number of studies remains limited, all currently published clinical trials have focused on TCM formulations that possess relatively robust preclinical evidence, making them research priorities. Notably, Scutellaria baicalensis (Huangqin), Coptis chinensis (Huanglian), Atractylodes macrocephala (Baizhu), and Glycyrrhiza uralensis (Gancao) emerged as the most frequently used herbs in the relevant formulas (Table 3). This finding aligns with a clinical data mining on medication rule analysis of TCM [205]. These results suggest that these herbs may play a pivotal role in managing CPT-11-induced diarrhea, providing critical evidence for future formula development and clinical translation assessment. In addition to direct clinical observations/efficacy confirmation trials, we also identified a run-in safety study designed to optimize the dosing regimen. This investigation specifically aims to evaluate the safety profile of XiaoChaiHu Decoction co-administered with CPT-11 through comprehensive pharmacokinetic monitoring under different intervention protocols. Although the relevant data have not yet been published, this pilot clinical study provides crucial preliminary evidence for identifying early safety concerns and enhancing the success rate of subsequent large-scale clinical trials. Furthermore, among accessible registered clinical trials, several terminated studies were identified, including Octreotide (NCT00006269), novel small-molecule compound (NCT00040391) and TCM formulation PHY906 (NCT00036517). Notably, PHY906 is a standardized formulation of HuangQin Decoction. Multiple clinical studies have demonstrated its efficacy in enhancing chemotherapy tolerance and reducing adverse effects, particularly diarrhea [206,207]. Preclinical evidence further supports PHY906’s potential in counteracting CPT-11-induced intestinal toxicity [151]. This terminated clinical trial was specifically designed to investigate the safety and tolerability of PHY906 combined with FOLFIRI chemotherapy, while simultaneously monitoring PHY906’s effects on CPT-11 pharmacokinetics and diarrhea severity. Although discontinued for undisclosed reasons, subsequent publications confirmed PHY906’s efficacy in controlling FOLFIRI-associated diarrhea, demonstrating a significant reduction in supplemental antidiarrheal medication use from 58.3% to 27.3% [208]. In summary, high-quality clinical evidence supporting TCM for managing CPT-11-induced diarrhea remains insufficient. Future research should expand clinical trials based on robust preclinical evidence and incorporate more detailed pilot studies to thoroughly evaluate intervention feasibility and mitigate potential risks. Such rigorous designs will provide a scientific foundation for subsequent confirmatory clinical trials.

### 6.2. Assessment of Clinical Translation Potential

As previously discussed, although clinical evidence supporting TCM for alleviating CPT-11-induced chemotherapy-associated diarrhea remains relatively limited, all published studies reporting positive outcomes have been substantiated by robust preclinical evidence. This underscores the critical role of preclinical research in facilitating clinical translation. HuangQin Decoction is a typical case of successful clinical translation. This classical Chinese herbal formula, composed of Scutellaria baicalensis (Huangqin), Paeonia lactiflora (Baishao), Glycyrrhiza uralensis (Gancao) and Ziziphus jujuba (Dazao), has been historically documented for alleviating abdominal cramps and diarrheal symptoms [225,226]. As evidenced in Table 2, HuangQin Decoction demonstrates a multifaceted therapeutic approach against CPT-11-induced intestinal toxicity, employing simultaneous mechanisms of intestinal epithelial repair, tight junction protein enhancement and anti-inflammatory action, thereby effectively addressing the key pathological processes of intestinal mucosal injury, tight junction disruption, and excessive release of pro-inflammatory cytokines (including TNF-α, IL-6, and IL-1β). Supported by these preclinical findings, Ren et al. conducted a clinical investigation involving 22 patients receiving HuangQin Decoction intervention. Their results demonstrated that prophylactic administration reduced the incidence of diarrhea (~50% reduction) and delayed its onset in patients undergoing the FOLFIRI chemotherapy regimen when compared with control groups [209]. Furthermore, PHY906, a quality-standardized formulation derived from HuangQin Decoction, has demonstrated significant clinical efficacy in an RCT. A phase I study involving 121 patients revealed that PHY906 administration reduced the incidence of CPT-11-associated diarrhea by 50% (*p* < 0.01), while maintaining the antitumor activity [208]. Based on these robust preclinical research data and their remarkable consistency with clinical observations, the pharmacological efficacy and safety profile of Huangqin Decoction have been scientifically validated. Consequently, this classical formulation was officially included in the “Catalog of Ancient Classical Formulas (Second Batch)” issued by the State Administration of Traditional Chinese Medicine of the People’s Republic of China in 2023. Such development not only facilitates clinical implementation but also exemplifies the critical role of preclinical research in advancing TCM modernization, thereby establishing an authoritative paradigm for evidence-based evaluation and translational application of traditional herbal formulations. In Table 2, we summarize the reported TCM compounds/extracts/monomers with their respective mechanisms for alleviating CPT-11-induced diarrhea in preclinical studies. Our analysis reveals that most TCM formulations demonstrate significant therapeutic potential and well-defined regulatory mechanisms. Notably, HuangQin Decoction, BanXiaXieXin Decoction, ShengJiangXieXin Decoction, GeGenQinLian Decoction, XiaoChaiHu Decoction, and TiaoPi AnChang Formula exhibit multi-pathway regulatory effects that precisely correspond to the complex pathological mechanisms underlying CPT-11-induced intestinal toxicity. It is particularly noteworthy that the TCM formulations that have demonstrated satisfactory clinical evidence to date (including HuangQin Decoction, BanXiaXieXin Decoction, and ShengJiangXieXin Decoction) share two critical characteristics: (1) robust preclinical pharmacological evidence encompassing both efficacy evaluation and mechanistic investigation, and (2) core herbal compositions containing high-frequency herbs such as *Scutellaria baicalensis* (Huangqin), *Coptis chinensis* (Huanglian), *Atractylodes macrocephala* (Baizhu), and *Glycyrrhiza uralensis* (Gancao). Based on these clues, we speculate that GeGenQinLian Decoction (containing *Coptis chinensis*, *Scutellaria baicalensis*, and *Glycyrrhiza uralensis*) and XiaoChaiHu Decoction (containing *Scutellaria baicalensis* and *Glycyrrhiza uralensis*) may possess significant clinical translation potential, warranting further evaluation through well-designed clinical trials.

### 6.3. Challenges of Clinical Translation

Although robust preclinical evidence facilitates successful clinical translation, the current translational process still faces multiple challenges. First, insufficient standardization in the quality control of TCM directly impacts the evaluation of clinical efficacy consistency and study reproducibility. Regarding translational strategies, organoid models may serve as a bridging technology to reduce the disparity between conventional animal models and human trials, thereby enhancing drug screening efficiency while reducing development costs. Regarding translational strategies, organoid models may serve as a bridging technology to reduce the disparity between conventional animal models and human trials, thereby enhancing drug screening efficiency while reducing development costs. Furthermore, clinical trial protocols must be rigorously standardized to ensure traceable and high-quality evidence generation. Additionally, compared with TCM formulations, clinical research on monomers/extracts remains markedly insufficient. Addressing these systemic challenges is critical for achieving high-quality clinical translation of TCM.

In conclusion, TCM can be used as the primary choice for the treatment of complex diseases, and effective TCM formulations should integrate both holistic and specific therapeutic advantages. The discovery of TCM formulas/components targeting multiple toxicological pathways of CPT-11 holds promise to address the shortcomings of current clinical therapeutic management. Furthermore, some treatment approaches based on diet or probiotics have also been gradually proposed (Table A1), which provide broader treatment options for CPT-11-induced diarrhea.

## 7. Discussion

As an effective anticancer drug, the balance between the therapeutic efficacy and gastrointestinal toxicity of CPT-11 has always been a significant challenge for clinicians and researchers. Here, we systematically summarize the intrinsic link between CPT-11 and gastrointestinal toxicity (with diarrhea as a typical representative), which can be mainly divided into two stages: (1) SN38 generation: CPT-11 undergoes various metabolic enzymes and transporters to generate SN38, which amplifies the cytotoxicity of CPT-11 (by several thousand fold). In particular, the metabolic transformation in the gut and the enterohepatic circulation significantly increase the exposure and duration of SN38, which are key factors contributing to the development of diarrhea [10,33,83]. (2) Induction of diarrhea: the amplified cytotoxicity, sufficient exposure duration, and adequate levels of SN38 undoubtedly exacerbate the stress on the gastrointestinal tract. Under these burdens, the intestinal barrier (including the gut microbiota and intestinal epithelial cells) is the first line of defense to be breached, which subsequently triggers cascade effects in intestinal inflammation, immune dysfunction, and metabolic disorders. These comprehensive factors eventually manifest as clinical symptoms of diarrhea [83,84,94,227]. Dedicated to mitigating the CPT-11-induced diarrhea, the recommended drugs in clinical management strategies have yet to demonstrate satisfactory outcomes. Scholars have endeavored to understand each aspect of CPT-11-induced diarrhea to identify specific therapeutic agents. Currently, several preclinical studies on single-target agents have made promising progress. However, this still deviates from the multi-link toxicological mechanisms of CPT-11-induced diarrhea. For many years, TCM has been regarded as a typical representative of multi-link synergistic action, and has been utilized in the treatment of various episodic and complex disorderss. In this review, we summarized the potential TCMS in preclinical studies for the treatment of CPT-11-induced diarrhea, including herbal compounds, extracts and active components. It was found that these TCMs not only regulate metabolic enzymes/transporters to prevent SN38 generation, but also repair the intestinal structure/function to maintain intestinal homeostasis, providing sustained protection against the two stages of CPT-11 gastrointestinal toxicity.

Although the multi-target regulatory advantages of traditional Chinese medicines (TCMs) are highly consistent with the intervention requirements for CPT-11-associated diarrhea, existing preclinical studies still have obvious limitations. Most available reports merely focus on the apparent pharmacodynamic effects of TCMs in alleviating diarrheal symptoms, while explorations of the underlying mechanisms lack systematicity and in-depth investigation. This is reflected in several critical aspects: (1) Investigations into TCM-mediated regulation of CPT-11-metabolizing enzymes and transporters have primarily focused on functional characterization (inhibition or activation of enzymatic activity/expression). However, the underlying molecular mechanisms, including nuclear receptors and transcriptional/translational regulation, remain to be fully elucidated. (2) Current research on TCM-mediated modulation of gut microbiota has primarily focused on macroscopic changes in microbial community structure and composition (α/β diversity, variation in relative abundance of phylum/genus). Nevertheless, deeper mechanistic investigations remain substantially underexplored, particularly regarding the identification of key functional microbiota with specific therapeutic effects and the molecular mechanisms underlying these functional microbiota–host interactions, such as microbiota/microbial metabolites–immune pathway crosstalk and microbiota/microbial metabolites–receptor signaling mechanisms. (3) The evaluation indicators for the anti-inflammatory effects of TCM are still insufficient, failing to capture the dynamic regulation of immunoinflammatory networks. (4) The molecular mechanism of TCM regulating metabolic network disorders is still insufficient, especially in the systematic identification and functional validation of predictive biomarkers using integrated multi-omics approaches.

At the clinical research level, the available trial evidence regarding TCM intervention for CPT-11-induced diarrhea exhibits notable deficiencies in terms of methodological quality control and strength of evidence. Specific shortcomings include: (1) Deficiencies in trial design, such as unclear randomization methods, imbalanced sample sizes between groups, insufficient patient numbers, and incomplete long-term follow-up data, all of which contribute to low-quality data sources. (2) Inadequate control of key confounding variables. Most enrolled patients received CPT-11-based combination chemotherapy regimens, making it difficult to exclude the synergistic effects of other cytotoxic drugs (including 5-FU, oxaliplatin) on diarrhea incidence. Consequently, this hinders the independent evaluation of TCM’s efficacy in alleviating CPT-11-specific diarrhea and impedes further exploration of its underlying pharmacological mechanisms.

In short, the treatment of CPT-11-related diarrhea requires a comprehensive consideration of both the efficacy and safety of therapeutic agents. TCM strategies offer a novel alternative. Following continued research and clinical practice, this holds promise to provide more effective and safer therapeutic options for patients with CPT-11-related diarrhea.

## 8. Conclusions

Mitigating irinotecan-associated diarrhea is an imperative prerequisite for achieving meaningful clinical benefit in patients receiving chemotherapy. This review systematically delineates the critical pathogenic events underlying irinotecan-induced diarrhea, tracing the sequence from the generation of the highly toxic metabolite SN38 mediated by metabolic enzymes and transporters to the subsequent perturbations in the intestinal microenvironment. Following the complete sequence of disease pathogenesis, we systematically summarize therapeutic interventions targeting each pathological injury node. Particular emphasis is placed on the therapeutic superiority of TCM. On one hand, it could modulate the key enzymes and transporters responsible for SN38 synthesis, thereby curtailing the accumulation of the toxic metabolite SN38 at the source. On the other hand, it also could attenuate the secondary intestinal damage provoked by SN38, encompassing restoration of the epithelial barrier, reconstruction of gut microbiota homeostasis, rectification of immune dysregulation, and suppression of excessive intestinal inflammation. Although TCM has demonstrated considerable potential in the prevention and treatment of CID, this field still lacks in-depth mechanistic elucidation coupled with sufficient high-quality clinical trial evidence, which greatly hinders the clinical translation and widespread clinical application of TCM.

In summary, irinotecan remains a cornerstone chemotherapeutic agent in clinical practice, and the establishment of comprehensive strategies for diarrhea prevention and management is essential to ensure its safe and widespread use. TCM, with its unique therapeutic advantages, offers a novel approach to balancing the antitumor efficacy of irinotecan against its intestinal toxicity. Continued efforts to deepen mechanistic investigations and promote high-quality clinical trials will facilitate the development of safer and more effective comprehensive interventions for patients with chemotherapy-induced diarrhea.

## Figures and Tables

**Table 1:** Small-molecule chemical drugs management for irinotecan-induced delayed diarrhea.

Name	Targets	Mechanism	Refs.
Remdesivir, Sofosbuvir	CES2	Reduce irinotecan hydrolysis: inhibit CES2 activity	[36]
TCH 3562	β-GUS	Reduce regeneration of SN-38: inhibit β-GUS activity	[45]
D-saccharic acid 1.4-lactone	β-GUS	Reduce irinotecan-induced mucosal damage: inhibit β-GUS activity	[137]
Amoxapine	β-GUS	Alleviate diarrhea: inhibit β-GUS activity	[138]
LE-77	CES2	Block irinotecan hydrolysis and reduce SN-38 accumulation: inhibit β-GUS activity	[133]
	FXR	Alleviate intestinal inflammation and preserve intestinal mucosal integrity: activate FXR activity	
Thalidomide	P-gp; MRP2	Reduce intracellular accumulation of irinotecan/SN38: inhibit P-gp and MRP2 transport capacity	[66,139]
Probenecid	MRP2	Inhibit biliary excretion to reduce irinotecan1/SN-38 exposure: inhibit the MRP2 transport capacity	[67]
Verapamil	P-gp	Inhibit biliary excretion to reduce irinotecan/SN-38 exposure: inhibit P-gp activity	[64]
Cyclosporin A	P-gp; MRP2	Inhibit biliary excretion to reduce irinotecan1/SN-38 exposure: inhibit P-gp and MRP2 transport capacity	[65]
Pentoxifylline	NA	Reduce lesions in intestinal mucosa: inhibit MPO, TNF-α and IL-1β overproduction	[140]
Thalidomide	NA	Reduce lesions in intestinal mucosa: inhibit TNF-α, IL-1, IL-6 and IL-1β overproduction	[66]
RDP58	NA	Reduce destruction of the intestinal mucosa: inhibit TNF-α, IFN-γ, and IL-12 overproduction	[141]
Celecoxib	NA	Reduce intestinal inflammation: inhibit COX-2 overproduction	[142]
IL-1Ra	NA	Reduce intestinal crypt apoptosis: enhance the basal level of IL-1 signaling	[134]
Darunavir	NLRP3	Reduce intestinal inflammation: inhibit NLRP3 activation	[143]
TU-100	TLR2	Enhance tight junction function: inhibit TLR2 activation	[116]
IAXO-102	TLR4	Reduce intestinal inflammation: inhibit TLR4 activation	[144]
AST-120	NA	Reduce intestinal exposure to irinotecan1/metabolites: adsorb free lumenal irinotecan1/metabolites	[135]
Activated charcoal	NA	Reduce intestinal exposure to irinotecan1/SN38: adsorb free lumenal SN38	[136]
Rifaximin	NA	Reduce irinotecan-induced mucosal damage: reduce SN38 exposure in intestine and promote an increase in beneficial microbiota	[145]

NA, not available.

**Table 2:** Traditional Chinese medicine management for irinotecan-induced delayed diarrhea.

Category	Name	Mechanism	Refs.
Formula	HuangQin Decoction (HQD)	Restore intestinal barrier dysfunction and the gut microbiota structure: reshape epithelial tight junction architecture, decrease the abundance of harmful bacteria (*Proteobacteria*), increase the abundance of beneficial bacteria (*Lactobacillus*)	[148]
		Protect the epithelial barrier and promote epithelial repair: increase the levels of nitric oxide (NO) in the gut, reduce the expression of proliferating cell nuclear antigen (PCNA)	[149]
		Reduce intestinal injury: regulate glycine, serine and threonine pathway	[103]
		Reduce intestinal injury: alter the metabolism of bile acids	[150]
		Restore the intestinal epithelium and reduce the intestinal inflammation: promote the regeneration of intestinal progenitor or stem cells and several Wnt signaling components, decrease the infiltration of neutrophils/macrophages and proinflammatory cytokine expression	[151]
		Repair intestinal barrier: inhibit endoplasmic reticulum stress through activating AMPK/mTOR-mediated autophagy	[152]
		Alleviate intestinal inflammatory injury: activate the p53/SLC7A11/GPX4 signaling pathway to inhibit ferroptosis	[153]
	ShengJiangXieXin Decoction (SJXXD)	Reduce intestinal inflammatory and maintain intestinal mucosal barrier integrity: improve apoptosis and necrosis of intestinal mucosal and functional cells	[154]
		Promote intestinal cell proliferation and reduce regeneration of SN-38: maintain intestinal stem cell function, inhibit intestinal apoptosis and β-GUS activity	[55]
		Restore the gut microbiota structure and intestinal barrier dysfunction: increase the composition and abundance of beneficial bacteria and reduce the β-GUS activity Reduce the intestinal inflammation and promote epithelial repair: inhibit the TLR4/NF-κB signaling pathway and promote mucin secretion in intestinal epithelial	[155]
		Reduce the intestinal inflammation: reduce inflammatory cytokines and neutropenia	[156]
		Maintain intestinal immune homeostasis: improve composition of T-lymphocytes and SIgA synthesis in intestinal tissue	[157]
		Inhibit the biliary excretion of irinotecan/SN-38: decrease hepatic MRP2 and P-gp expression, and alter the activities of CES and UGT	[71]
		Reduce intestinal SN38 exposure: increase hepatic UGT1A1 expression and reduce β-GUS activity in the intestine	[158]
	XiaoChaiHu Decoction (XCHD)	Reduce the intestinal inflammation: reduce the levels of pro-inflammatory cytokines (TNF-α, COX-2, PGE2, IL-1β, IL-6) and improve the levels of anti-inflammatory cytokines (IL-10, IL-15)	[159]
		Inhibit the biliary excretion of irinotecan/SN-38 and accelerate the elimination: reduce inflammatory cell infiltrate	[160]
		Decrease the concentrations of SN38 and restore gut microbiota balance: inhibit β-GUS activity and improve community abundances of beneficial bacteria (*Firmicutes* and *Bacteroidota*)	[53]
		Inhibit both biliary and intestinal excretions of irinotecan, SN-38, and SN-38G	[161]
		Reduced intestinal SN38 exposure: increase intestinal UGTs expression and activity	[72]
		Inhibit the efflux transporters (ABCB1, ABCC2, ABCG2) and β-GUS activity	[162]
	BanXiaXieXin Decoction (BXXXD)	Restore gut microbiota balance: restore the structure and number of gut microbiota and inhibit pathogen growth while promoting probiotic viability	[159]
		Reduce the intestinal inflammation: inhibit TLR4/NF-κB signaling pathway	[163]
	DaBuPi Decoction (DBPD)	Restore gut microbiota balance and inhibit the biliary excretion of irinotecan/SN-38: decrease the abundance of harmful bacteria *(Hypopseudomonas*, *Bacteroides*, *Erysipelothrix*), increase the abundance of beneficial bacteria (*Lactobacillus*), decrease β-GUS activity and MRP2 protein expression	[59]
	GeGenQinLian Decoction (GGQLD)	Restore the intestinal barrier function: activate the Keap1/Nrf2 pathway and enhance the expression of tight junction proteins (ZO-1,occludin) Reduce the intestinal inflammation: inhibit the PI3K/AKT/NF-κB signaling pathway	[164]
		Protect the cellular proliferation and ameliorate diarrhea: inhibit the PI3K/AKT/NF-κB axis	[165]
	GanCaoXieXin Decoction (GCXXD)	Restore gut microbiota balance: increase the abundance of beneficial bacteria (*Bacteroides*, *Muribaculaceae*)	[166]
	HuangLianJieDu Decoction (HLJDD)	Promot epithelial repair: maintain intestinal crypt renewal, activate the Wnt/β-catenin pathway	[167]
	TiaoPiAnChang (TPAC)	Restore the gut microbiota structure and reduce intestinal SN38 exposure: restore the abundance, evenness, and diversity of intestinal microbiota Reduce the intestinal inflammation and promote epithelial repair: inhibit the TLR44/NF-κB/NLRP3 signaling pathway	[168]
	GuangHuoXiang Granules (GHXKL)	Restore the gut microbiota structure: decrease the abundance of harmful bacteria and increase the abundance of beneficial bacteria (*Lactobacillus*)	[169]
	FuZiLiZhong Pill (FZLZP)	Reduce the intestinal inflammation: inhibit NLRP-3 inflammasome activation	[170]
	WeiChangAn pill (WCAP)	Reduce the intestinal inflammation and SN38 exposure: inhibit the TLR4/NF-κB signaling pathway and β-GUS activity	[171]
	SiShen Pill (SSP)	Reduce the intestinal inflammation and SN38 exposure: reduce the levels of pro-inflammatory cytokines (TNF-α, IL-1β) and inhibit β-GUS activity	[172]
	ShuBuWenShenGuChang (SBWSGC)	Reduce the intestinal inflammation: inhibit the TLR4/NF-κB signaling pathway	[173]
	HeZhongFang (HZF)	Reduce the intestinal inflammation: inhibit AIM2 inflammasomes activation	[174]
	TangCao table (TCT)	Reduce inflammatory cell infiltration and repair intestinal mucosal injury	[175]
	ShenLinBaiZhuSan (SLBZS)	Reduce the intestinal inflammation	[176]
		Reduce the intestinal inflammation: inhibit the aberrant activation of inflammatory signaling pathways, including PI3K/AKT, MAPK, and NF-κB	[177]
	JianPiYiQiFang (JPYQ)	Improve diarrhea based on clinical observation	[178]
	GuBenYiChang (GBYC)	Improve diarrhea based on clinical observation	[179]
	***Brucea javanica* Oil Emulsion**	Mitigate inflammation and preserve intestinal barrier function: Inhibit the cGAS-STING pathway	[180]
Extract	Chloroform extract of GGQLT	Restore intestinal barrier dysfunction: reduce inflammatory cytokines and inhibit oxidative stress	[181]
	Ling Zhi-8	Mitigate intestinal damage: restore claudin-1 expression in intestinal cells	[182]
	Total Flavonoids of Glycyrrhiza uralensis	Restore the gut microbiota dysbiosis: reduce the abundance of β-GUS producing bacteria (Escherichia coli and Clostridium) and increase the abundance of SCFs-producing bacteria (Roxiella spp.)	[183]
	Green tea extract	Decrease the production of SN-38: decrease the abundance of β-GUS-producing bacteria Accelerate inactivation and elimination of SN38: increase UGT1A1 levels	[54]
	G. lucidum polysaccharides	Increase number of neutrophils and lymphocytes	[184]
	St. John’s wort	Down-regulate intestinal pro-inflammatory cytokines and inhibit intestinal epithelial apoptosis	[185]
	Flavonoid of Bauhinia forficata leaves	Reduce intestinal mucosal inflammation	[186]
	Water extract of Mume Fructus	Reduce intestinal mucosal inflammation: reduce the levels of pro-inflammatory cytokines (TNF-α, IL-1β)	[187]
	Total Flavonoids of Glycyrrhiza uralensis	Reduce the fecal metabolic disorders: reduce the fluctuation of amino acid metabolism and purine metabolism	[54]
Monomer	Curcumin	Reduce intestinal cell apoptosis: inhibit NF-κB activation and oxidative stress	[188]
	Phytocannabinoids	Decrease intestinal epithelial permeability: inhibit the production of pro-inflammatory cytokines	[189]
	Hesperidin	Promote epithelial repair: reduce inflammatory cytokines, oxidative stress and cell apoptosis	[190]
	Berberine	Decrease the production of SN-38: inhibit β-GUS activity and β-GUS-producing bacteria	[52]
		Alleviate gastrointestinal dysfunction: inhibit ALOX15-mediated ferroptosis and downregulate glutathione peroxidase 4 (Gpx4) and SLC7A11 expression	[191]
	Anthocyanins	Reduce intestinal mucositis: inhibit the mTOR pathway	[192]
	Luteolin	Decrease inflammatory mediator expression: upregulate intestinal PPARγ expression Reduce the intestinal inflammation: reduce the levels of oxidative stress (ROS, MPO) and inflammatory cytokines (TNF, IL-1β and IL-6)	[193]
	Carvacrol	Reduce the intestinal inflammation: activate the TRPA1 receptor	[129]
	Dehydrocostus lactone	Reduce the intestinal mucositis: inhibit the TLR4/NF-κB/NLRP3 signaling pathway	[194]
	Andrographolide	Promote DNA damage repair: downregulate dsDNA–cGAS–STING signaling	[195]
	Baicalin	Inhibit the biliary excretion of irinotecan/SN-38: inhibit hepatic OATP expression	[191]
	Oroxylin A	Reduce intestinal SN38 production: inhibit intestinal CES2 activity	[37]
	Schisandrin	Reduce intestinal SN38 production: inhibit intestinal CES2 activity	[196]
	Genistein	Inhibit the biliary excretion of irinotecan/SN-38: inhibit intestinal MRP2 activity	[70]
	Artesunate	Suppress cellular senescence: inhibited mTOR activity	[197]
	Brusatol	Mitigate intestinal damage: inhibit the cGAS-STING pathway and modulation of intestinal flora	[127]
	Hesperidin	Improve intestinal barrier integrity: modulate the gut microbiota and inhibit the IL-17 signaling pathway	[198]
	Saikosaponin-d	Protect intestinal epithelial integrity: inhibit the TAK1/NF-κB pathway	[199]
	Tectorigenin	Alleviate intestinal inflammation: activate the Nrf2/Keap1 signaling pathway	[200]

**Table 3:** Clinical Trials of Traditional Chinese Medicine for Irinotecan-Induced Diarrhea.

Type of Clinical Trials	TCM	Herbal Composition	Case Distribution	Chemotherapy Regimens	Intervention Duration	Outcome Measures	Refs.
RCT (Completed)	HuangQin Decoction (HQD)	Huangqin (20 g), Baishao (15 g), Gancao (15 g), Dazao (30 g)	Metastatic colorectal cancer (*n* = 44)	FOLFIRI	Hospital preparation, 20 mL three times daily, starting 1 day before chemotherapy for 28 consecutive days	Diarrhea incidence, diarrhea grade, mean time to diarrhea onset, diarrhea duration, other clinical manifestations (abdominal pain, bloody stool, fever, leukocytosis, liver dysfunction)	[209]
RCT (Completed)	PHY906	Huangqin, Baishao, Gancao, Dazao	Colorectal cancer (*n* = 17)	IFL	Standardized formulation, dose escalation followed Fibonacci sequence (1.2 g, 2.4 g, and 3.6 g) for 48 consecutive days	Diarrhea incidence, safety evaluation (nausea/vomiting, anorexia, abdominal distension/pain, neutropenia, leukopenia severity)	[208]
RCT (Completed)	JiaWeiHuangQin Decoction (JWHQD)	Huangqin (20 g), Baishao (15 g), Dangshen (20 g), Jiangbanxia (10 g), Shengmaiya (15 g), Gancao (15 g)	Colorectal cancer (*n* = 72)	FOLFIRI	Hospital preparation, 1 dose daily, starting 1 day before chemotherapy for 7 consecutive days	Diarrhea incidence, other clinical manifestations (myelosuppression, nausea/vomiting, anorexia)	[210]
RCT (Completed)	BanXiaXieXin Decoction (BXXXD)	Banxia (10 g), Haungqin, Ganjiang, Renshen, Gancao (9 g), Huanglian (3 g), Dazao (6 g)	Gastric cancer (*n* = 3), colorectal cancer (*n* = 26)	NR	BanXiaXieXin Decoction granules (Guangdong Yifang Pharmaceutical Co., Ltd., Foshan, China), 1 dose daily for 28 consecutive days	Diarrhea grading (CTCAE v5.0), T-cell subsets (CD3+, CD4+, CD8+, CD4+/CD8+), TCM syndrome score (Abdominal distension/pain, nausea/vomiting, appetite, mental/physical status), Karnofsky Performance Status (KPS)	[211]
RCT (Completed)	BanXiaXieXin Decoction (BXXXD)	Banxia (12 g), Haungqin, ChuanHouPu, ChenPi, Dangshen, Baidoukou (10 g), Gancao (5 g)	Colorectal cancer (*n* = 23), lung cancer (*n* = 12), gastric cancer (*n* = 10), Ovarian cancer (*n* = 3), metastatic omental cancer (*n* = 2)	FOLFIRI, FOLFIRI, XELIRI, IROX or IP-NDP	200 mL decoction (boiled water extraction) administered as 100 mL twice daily, starting 1 day before chemotherapy for 15 consecutive days	Diarrhea incidence, nausea/vomiting, leukopenia severity	[212]
RCT (Completed)	BanXiaXieXin Decoction (BXXXD)	Ganjiang (9 g), Renshen (9 g), Huanglian (3 g), Huangqin (9 g), Banxia (12 g), Gancao (9 g), Dazao (12 pieces)	Colorectal cancer (*n* = 14), small cell lung cancer (*n* = 17)	FOLFIRI, XELIRI, IROX, IP or IC	Hospital preparation, three times daily, starting 1 day before chemotherapy for 14 consecutive days	Diarrhea incidence, diarrhea grading, mean time to diarrhea onset, diarrhea duration, Bristol Stool Form Scale (BSFS), KPS, safety evaluation (myelosuppression, hepatic/renal impairment, nausea/vomiting, anorexia, abdominal distension/pain)	[213]
RCT (Completed)	BanXiaXieXin Decoction (BXXXD)	Banxia (12 g), Shengjiang (10 g), Huangqin (10 g), Dangshen (12 g), Huanglian (5 g), Gancao (5 g), Dazao (12 g)	Colorectal cancer (*n* = 46), small cell lung cancer (*n* = 9)	FOLFIRI	Hospital preparation, 1 dose daily, starting 1 day before chemotherapy for 5 consecutive days	Diarrhea incidence, diarrhea grading (JCOG Clinical Trial Protocol Template), leukopenia severity, nausea and vomiting	[214]
RCT (Completed)	BanXiaXieXin Decoction (BXXXD)	Banxia (12 g), Ganjiang (9 g), Huangqin (9 g), Dangshen (9 g), Huanglian (3 g), Gancao (9 g), Dazao (12 g)	Extensive-stage small cell lung cancer (*n* = 36)	IP	Banxia Xiexin Decoction granules (Sichuan New Green Pharmaceutical Technology Development Co., Ltd., Chengdu, China), twice daily, starting 1 day before chemotherapy for 5 consecutive days	Diarrhea grading (JCOG Clinical Trial Protocol Template), leukopenia severity	[215]
RCT (Completed)	BanXiaXieXin Decoction (BXXXD)	Banxia (12 g), Ganjiang (9 g), Huangqin (9 g), Dangshen (9 g), Huanglian (3 g), Gancao (9 g), Dazao (12 g)	Extensive-stage small cell lung cancer (*n* = 36)	IP	Hospital preparation, 1 dose daily, starting 1 day before chemotherapy for 5 consecutive days	Diarrhea grading (JCOG Clinical Trial Protocol Template), leukopenia severity	[215]
RCT (Completed)	Hangeshashin-to	Banxia (15 g), Ganjiang (9 g), Huangqin (9 g), Dangshen (9 g), Huanglian (3 g), Gancao (9 g), Dazao (4 g), Renshen (9 g)	Metastatic colorectal cancer (*n* = 29)	FOLFIRI	Hangeshashin-to (Tsumura & Co., Tokyo, Japan), 2.5 g three times daily, starting 3 days before chemotherapy for 21 consecutive days	Diarrhea grading (CTCAE v3.0), safety evaluation (neutropenia, leukopenia severity)	[216]
RCT (Completed)	Hangeshashin-to	Banxia (15 g), Ganjiang (9 g), Huangqin (9 g), Dangshen (9 g), Huanglian (3 g), Gancao (9 g), Dazao (4 g), Renshen (9 g)	Lung cancer (*n* = 41)	IP	Hangeshashin-to (Tsumura & Co.): 2.5 g three times daily (tid), starting 3 days before chemotherapy for 14 consecutive days	Diarrhea grade (CTCAE v3.0), stool characteristics, bowel frequency, abdominal pain severity, bloody stool presence,	[217]
RCT (Completed)	JiaWeiBanXiaXieXin Decoction (JWBXXXD)	Banxia (12 g), Ganjiang (10 g), Huangqin (10 g), Dangshen (10 g), Chenpi (10 g), Baidoukou (10 g), Chuanpu (10 g), Gancao (9 g)	Colorectal cancer (*n* = 8), lung cancer (*n* = 7), gastric cancer (*n* = 6), ovarian cancer (*n* = 2), metastatic omental cancer (*n* = 1), mesenteric metastatic carcinoma (*n* = 1)	FOLFIRI, FOLFIRI, XELIRI, IROX or IP-NDP	Hospital preparation, 1 dose daily, starting 1 day before chemotherapy for 5 consecutive days	Diarrhea grading (JCOG Clinical Trial Protocol Template), nausea/vomiting, leukopenia severity,	[218]
RCT (Completed)	JiaWeiBanXiaXieXin Decoction (JWBXXXD)	Banxia (10 g), Baizhu (10 g), Fulin (10 g), Huangqin (10 g), Huanglian (10 g), Ganjiang (10 g), Jupi (10 g), Jineijin (20 g), Gancao	Colorectal cancer (*n* = 59)	NR	150 mL decoction (boiled water extraction) administered as 75 mL twice daily, starting 1 day before chemotherapy for 7 consecutive days	Diarrhea severity (ECOG CTC v3.0), serum β-GD activity, myelosuppression (CTCAE v3.0), Karnofsky Performance Status (KPS)	[219]
RCT (Completed)	ShengJiangXieXin Decoction (SJXXD)	Shengjiang (12 g), Banxia (9 g), Dangshen (9 g), Ganjaing (3 g), Huangqin (9 g), Huanglian (3 g), Dazao (12 g), Gancao (6 g)	Small cell lung cancer (*n* = 56)	NR	Hospital preparation, 1 dose daily, starting 1 day before chemotherapy for 28 consecutive days	Diarrhea incidence, diarrhea grading (CTCAE v5.0), mean time to diarrhea onset, diarrhea duration, neutropenia grade, safety evaluation (CBC, hepatic/renal function, coagulation, ECG	[220]
RCT (Completed)	ShengJiangXieXin Decoction (SJXXD)	Shengjiang (12 g), Banxia (9 g), Dangshen (9 g), Ganjaing (3 g), Huangqin (9 g), Huanglian (3 g), Dazao (12 g), Gancao (9 g)	Colorectal cancer (*n* = 68)	FOLFIRI	Hospital preparation, 1 dose daily, starting 2 day before chemotherapy for 10 consecutive days	Diarrhea severity (ECOG CTC v3.0 criteria), serum T-cell subsets, NK cell activity, Karnofsky Performance Status (KPS)	[221]
RCT (Completed)	ShenLinBaiZhu Powder (SLBZP)	Dangshen (15 g), Baizhu (10 g), Fulin (15 g), Gancao (3 g), Lianzi (15 g), Yiyiren (15 g), Sahren (3 g), Baindou (15 g), Jiegen (5 g), Huaishanyao (15 g)	Colorectal cancer (*n* = 60)	FOLFIRI	Hospital preparation, 1 dose daily, starting 2 day before chemotherapy for 28 consecutive days	Diarrhea grading (CTCAE v5.0), Karnofsky Performance Status (KPS), T-cell subsets (CD3+, CD4+, CD8+, CD4+/CD8+), other clinical manifestations (myelosuppression, liver dysfunction, nausea/vomiting)	[222]
RCT (Completed)	JiaWeiShenLinBaiZhu Powder (JWSLBZP)	Shengshaisheen (30 g), Fulin (15 g), Baizhu (15 g), Baidou (15 g), Chenpi (20 g), Lianzi (15 g), Gancao (3 g), Shanyao (20 g), Sharen (15 g), Yiyiren (30 g), Jiegeng (15 g), Huanqi (30 g)	Colorectal cancer (*n* = 46)	FOLFIRI	Hospital preparation, 1 dose daily, starting 2 day before chemotherapy for 28 consecutive days	Diarrhea incidence, diarrhea grading (CTCAE v5.0), mean time to diarrhea onset, Karnofsky Performance Status (KPS), other clinical manifestations (myelosuppression, liver dysfunction)	[223]
RCT (Completed)	TongXieYaoFang (TXYF)	Banxia (30 g), Baishao (20 g), Chenpi (15 g), Fangfeng (10 g)	Colorectal cancer (*n* = 80)	FOLFIRI	300 mL decoction (boiled water extraction) administered as 150 mL twice daily, starting 1 day before chemotherapy for 20 consecutive days	Diarrhea severity (ECOG CTC v3.0), serum β-GD activity, myelosuppression (CTCAE v3.0), Karnofsky Performance Status (KPS)	[224]
Run-in safety study (Completed)	XiaoChaiHu Decoction (XCHD)	NR	Malignant tumors (*n* = 24)	FOLFIRI	XiaoChaiHu Decoction granules (Yan Chai Hospital Pharmaceutical Institute (Hong Kong, China) Ltd.), 9 g once daily, starting 3 days before chemotherapy for 5 consecutive days	Diarrhea grade (CTCAE v5.0), fecal occult blood	NA
RCT (Recruiting)	XiaoChaiHu Decoction (NCT06055179)	NR	Malignant tumors	FOLFIRI, mXELIRI, CapIRI, FOLFOXIRI	XiaoChaiHu Decoction granules (Yan Chai Hospital Pharmaceutical Institute (Hong Kong) Ltd.), 9 g once daily, starting 3 days before chemotherapy for 5 consecutive days	Diarrhea grade (CTCAE v5.0), fecal occult blood	NA
RCT (Terminated)	PHY906 (NCT00036517)	NR	Colorectal cancer (*n* = 80)	NR	NR	NR	NA
RCT (Not recruiting)	ShengJiangXieXin Decoction (ChiCTR1800018490)	NR	Small cell lung cancer (*n* = 64)	NR	NR	Diarrhea incidence, neutrophil reduction	NA
RCT (Recruiting)	Berberine (ChiCTR2300078353)	NR	Colorectal cancer (*n* = 80)	FOLFIRI, FOLFIRI, XELIRI, IROX or IP-NDP	NR	Diarrhea incidence, adverse event rate, Progression-Free Survival (PFS), Objective Remission Rate (ORR), Disease Control Rate (DCR), Time To Progress (TTP), Gut microbiota, Cytokine	NA

NR, not reported; NA, not available.

## Data Availability

No new data were created or analyzed in this study. Data sharing is not applicable to this article.

## Abbreviations

The following abbreviations are used in this manuscript:

CPT-11	Irinotecan
TCM	Traditional Chinese medicine
SN38	7-ethyl-10-hydroxy-camptothecin
SN38G	SN38 glucuronidate
BES	Butyrylcholinesterases
CES	Carboxylesterases
UGT1A1	Uridine diphosphate-glucuronosyltransferase 1A1
β-GUS	β-glucuronidase
OATPs	Organic anion transporting polypeptides
ABC	ATP-binding cassette transporters
CID	Chemotherapy-induced diarrhea
TJs	Tight junctions
SCFAs	Short-chain fatty acids
IAA	Indole acetic acid
IAld	Indole formaldehyde
IA	Indole acrylic acid
ILA	Indole lactic acid
BAs	Bile acids
DAMPs	Damage-associated molecular patterns
PRRs	Pattern recognition receptors
TLRs	Toll-like receptors
NLRs	NOD-like receptors
TRPA1	Transient receptor potential ankyrin 1
SIgA	Secretory immunoglobulin A
RR	Relative risk
CI	Confidence interval
RCT	Randomized controlled trial

## Appendix A

**Table A1:** Small-molecule chemical drug management for irinotecan-induced delayed diarrhea.

Category	Name	Mechanism	Refs.
Antibiotics	Saccharomyces boulardii	Reduce intestinal mucositis	[228]
Dietary supplements	Vitamin A	Protect the intestinal barrier: inhibit immune infiltration and reactive enteric gliosis	[229]
	Glutamine	Improve intestinal immune function and reduce intestinal SN38 exposure: regulate immune cells and inhibit the β-GUS activity	[230]
	Butyrate	Improve epithelial permeability and inhibit β-GUS activity	[231]
	S-adenosylmethionine	Improve intestinal barrier dysfunction and inhibit the increase in intestinal Proteobacteria abundance induced by irinotecan: inhibit ROS production and activation of the p38 MAPK/p65 NF-κB/MLCK/MLC signaling pathway	[87]
Probiotics	Streptomycin	Reduce intestinal irinotecan/SN-38 exposure: reduce CES activity and increase UGT activity	[48]
	Bifidobacterium longum SX-1326	Restore gut microbiota balance and alleviate intestinal mucosa: increase levels of beneficial bacteria (*Ruminococcus and Pilospirillum*), Downregulate TLR4/MyD88/NF-κB signaling pathway	[232]
	Escherichia coli Nissle 1917	Restore gut microbiota balance and alleviate intestinal mucosa: increase the diversity of gut microbiota and decreased intestinal epithelial permeability	[86]
	Bifidobacterium longum subsp. longum 51A	Reduce intestinal mucositis: increase sIgA production and reduce the composition of harmful bacteria	[233]
	Paraprobiotic Enterococcus faecalis EC-12	Reduce intestinal mucositis: reduce the infiltration of neutrophils and inflammatory cytokines	[234]
	Bifidobacterium longum	Reduce intestinal mucositis: inhibit the intestinal TNF-α and IL-1β levels	[235]
		Alleviate intestinal mucositis: inhibit the STING/NF-κB pathway	[236]
